# Asthma and incident coronary heart disease: an observational and Mendelian randomisation study

**DOI:** 10.1183/13993003.01788-2023

**Published:** 2023-11-30

**Authors:** Carlos A. Valencia-Hernández, Fabiola Del Greco M, Varun Sundaram, Laura Portas, Cosetta Minelli, Chloe I. Bloom

**Affiliations:** 1 National Heart and Lung Institute, Imperial College London, London, UK; 2 Institute for Biomedicine, Eurac Research, Bolzano, Italy; 3 Louis Stokes Cleveland Medical Center, Case Western Reserve University, Cleveland, OH, USA; 4 Oxford Big Data Institute, University of Oxford, Oxford, UK

## Abstract

**Background:**

Observational studies suggest asthma is a risk factor for coronary heart disease (CHD) and sex modifies the risk, but they may suffer from methodological limitations. To overcome these, we applied a “triangulation approach”, where different methodologies, with different potential biases, were leveraged to enhance confidence in findings.

**Methods:**

First, we conducted an observational study using UK medical records to match asthma patients 1:1, by age, sex and general practitioner (GP) practice, to the general population. We measured the association between asthma and incident CHD (myocardial infarction: hospitalisation/death) by applying minimal sufficient adjustment: model 1, smoking, body mass index, oral corticosteroids, atopy and deprivation; model 2, additionally adjusting for healthcare behaviour (GP consultation frequency). Second, we conducted a Mendelian randomisation (MR) study using data from the UK Biobank, Trans-National Asthma Genetic Consortium (TAGC) and Coronary Artery Disease Genome-wide Replication and Meta-analysis consortium (CARDIoGRAM). Using 64 asthma single nucleotide polymorphisms, the effect of asthma on CHD was estimated with inverse variance-weighted meta-analysis and methods that adjust for pleiotropy.

**Results:**

In our observational study (n=1 522 910), we found asthma was associated with 6% increased risk of CHD (model 1: HR 1.06, 95% CI 1.01–1.13); after accounting for healthcare behaviour, we found no association (model 2: HR 0.99, 95% CI 0.94–1.05). Asthma severity did not modify the association, but sex did (females: HR 1.11, 95% CI 1.01–1.21; males: HR 0.91, 95% CI 0.84–0.98). Our MR study (n=589 875) found no association between asthma and CHD (OR 1.01, 95% CI 0.98–1.04) and no modification by sex.

**Conclusions:**

Our findings suggest that asthma is not a risk factor for CHD. Previous studies may have suffered from detection bias or residual confounding.

## Tweetable abstract

**Our study using a triangulation approach, real-world data and genetic epidemiology found no association between asthma and coronary heart disease, in contrast to earlier studies** https://bit.ly/3FuZHaP

## Introduction

Asthma is thought to be a risk factor for coronary heart disease (CHD) due to numerous population-based observational studies [1–15]. Several hypotheses have been postulated, including shared risk factors (obesity, cigarette smoke and pollution), reduced lung function [16] and inhaled bronchodilators (shown to exert cardiotoxic effects in COPD patients) [17]. However, the association thus far observed may not be causal.

A meta-analysis of 30 observational studies by Hua *et al.* [18] reported a 39% increased risk of myocardial infarction and 35% increased risk of cardiovascular mortality associated with asthma. However, the reliability of their conclusion was reduced by study limitations, including small sample size, lack of adjustment for key confounders (*e.g.* smoking and obesity), high study heterogeneity and the use of decades-old data when diagnostic capabilities differed from current medicine. More contemporary, larger studies, using US administrative data (0.2 million asthma adults) [6], the Taiwanese national health-check programme (0.4 million adults, >14 000 asthma) [10] and the Copenhagen General Population Study (0.1 million adults, >5000 asthma) [8], all found an association of asthma with CHD, but observed opposing risks for different subgroups. The meta-analysis found sex to be a significant modifier, with only females at increased risk; in contrast, the large Taiwanese study found males were more at risk of cardiovascular mortality [10]. The US study found allergy to be a risk factor for CHD, but allergic asthma was not [6]. The Copenhagen study observed an increased risk with asthma only for smokers [8]. These discrepancies between studies reduce our confidence that the observed association is causal, raising suspicion of bias or confounding. Furthermore, a recent Mendelian randomisation (MR) study found lung volume, rather than lung obstruction, is causally associated with CHD [19].

In this study, to overcome some of these limitations, we have used a triangulation approach that integrates different epidemiological methods and data resources to provide more reliable findings. This integration of evidence from alternative approaches, with differing potential biases, is applied in aetiological epidemiology to improve causal inference [20]. First, we conducted an observational study using a nationwide dataset, the largest observational asthma–cardiovascular disease study to date, and we considered several confounders, including healthcare behaviour, which could cause a detection bias. Patients with asthma have been found to display differential healthcare behaviour compared with the general population, including enhanced healthcare seeking [21]. Second, we performed a MR study, using genetic data from large available datasets. MR is not affected by issues of classical confounding and reverse causation (*i.e.* CHD increasing the risk of asthma, rather than vice versa) and can therefore provide stronger evidence of a possible causal effect when its assumptions are satisfied.

## Methods

### Observational study

#### Data resources

We used the Clinical Practice Research Datalink (CPRD), a nationwide database of routinely collected information from general practitioner (GP) practices covering ∼20% of the UK, individually linked to Hospital Episode Statistics (English hospital admission data) and Office of National Statistics mortality data.

#### Study population and design

Adults (≥18 years) contributing data between 1 January 2004 and 2019, with at least 1 year of data before study entry, were eligible for inclusion. Follow-up ended at the earliest of 1 January 2019, last data collection date or death. We conducted a matched cohort: matching asthma patients 1:1, by birth year, sex and GP practice, to the “general population” who were randomly selected from all other patients (supplementary figure S1).

Asthma was defined as the presence of at least two validated asthma CPRD Read codes [22], ≤3 years from study entry (supplementary table S1). As the outcome was incident CHD, patients with a history of CHD at cohort entry were excluded, as well as those with known COPD.

#### Outcomes and covariates

The outcome, incident CHD, was identified using International Classification of Diseases, 10th Revision codes I21–I23, hospital admission or death.

Model covariates included variables considered *a priori* to be possible confounders: body mass index (BMI), smoking history (current smoker/ex-smoker/never-smoker), socioeconomic status (Index of Multiple Deprivation), atopy and GP consultations (see directed acyclic graphs in supplementary figure S2).

Asthma severity was described by asthma medication prescribed in the year before study entry: short-acting β_2_-agonists (SABA), inhaled corticosteroids (ICS) and ICS with additional (“add-on”) asthma medication (long-acting bronchodilators or leukotriene receptor antagonists). Asthma exacerbations were defined as a short course of oral corticosteroids (OCS) or emergency department/hospital admission for asthma.

The “GP consultations” variable was used as a proxy for healthcare behaviour and defined as the number of GP practice consultations in the year before study entry (excluding administrative codes); codes included blood pressure, blood tests, disease monitoring and vaccinations. Allowing one consultation per day, the variable was categorised into quartiles. Frequent GP consultations are likely to be associated with conditions that require regular health checks (*e.g.* asthma and hypertension), health anxiety or frailty. Healthcare behaviour was considered *a priori* as a confounder (supplementary figure S2a) as it may lead to detection bias due to increased/earlier symptom reporting (*e.g.* breathlessness, chest pain and palpitations). We cannot exclude that healthcare behaviour might instead represent a mediator (supplementary figure S2b), but even in this case we wished to adjust for it as we are interested in the direct effect of asthma on CHD, not an indirect effect through increased checking of symptoms. In this setting, healthcare behaviour measured at baseline cannot be a collider as the outcome is a new diagnosis of CHD. We evaluated the variable as described in the Statistical analysis section.

#### Statistical analysis

To estimate the association between asthma and incident CHD, we used multivariable stratified Cox proportional hazard models, stratified by matched set, with time in study as the timescale. The assumptions of proportional hazards were met (tested by visualising the Schoenfeld residuals).

There were two main models: model 1 was adjusted for BMI, socioeconomic status, smoking, atopy and asthma exacerbations; model 2 was additionally adjusted for GP consultations (supplementary figure S2a). Additionally, we adjusted model 2 for asthma severity.

For covariates with >10% missing data (BMI and smoking), we imputed missing values using multiple imputation by chained equations; imputation models included all covariates and the outcome, with the results of 10 imputed datasets pooled using Rubin's rules.

We evaluated the GP consultations variable in several ways. 1) Describing patient characteristics by quartile. 2) Using Cox models to assess the association between asthma and two outcomes: a) consultations for minor ailments (sore throat, cold sore, coryzal symptoms, headaches and tiredness) and b) all-cause mortality. These two extreme outcomes were chosen to assess if frequent GP consultations were associated with all aspects of healthcare seeking or only those of a more serious nature. We then adjusted both models for GP consultations to assess any confounding effects. 3) Using multinomial logistic regression to assess if people with asthma had an increased association with GP consultations even prior to their diagnosis.

We fitted interactions to model 2 to investigate modification by sex, smoking (ever/never smoked) and eosinophil count (≤300 or >300 cells·µL^−1^ ≤3 years of study entry); these were compared with the main model using likelihood ratio tests. Lastly, stratified analyses were performed where an interaction was present and for asthma severity. All analyses were conducted using Stata version 17 (StataCorp, College Station, TX, USA).

### MR study

We used MR to estimate the casual effect of asthma on CHD risk. This approach exploits genetic data to derive an estimate of the effect of the exposure (asthma) on the outcome (CHD) that is not affected by classical confounding or reverse causation. Genetic variants (single nucleotide polymorphisms (SNPs)) associated with the exposure (asthma) are selected as its proxies (see SNP selection section). For each SNP, two sets of genetic association estimates are obtained for its association with asthma (“GX” estimate) and CHD (“GY” estimate), and the estimate of the the causal effect of interest of asthma on CHD is then derived from GX and GY (see supplementary figure S3 and the MR analysis section).

#### GX study populations

GX estimates were obtained from the UK Biobank (UKB) and Trans-National Asthma Genetic Consortium (TAGC).

UKB is a study of 500 000 participants from the UK, age 40–69 years. From the 488 377 participants with genetic data, 460 186 were of White European ancestry, of which 344 742 had information on asthma: 49 008 cases (self-reported doctor-diagnosed asthma; supplementary table S2) and 295 734 controls (no asthma) [23].

TAGC is an international consortium of 56 genome-wide association studies (GWASs) with a total of 142 486 individuals of diverse ancestries. We only included those of White European ancestry: 19 954 cases (asthma: doctor diagnosed and/or from standardised questionnaires; supplementary table S2) and 107 715 controls (no asthma).

#### GY study populations

GY estimates were obtained from UKB and the Coronary Artery Disease Genome-wide Replication and Meta-analysis consortium (CARDIoGRAM) [24].

From the 488 377 UKB participants with genetic data, we included 405 570 with available information for self-reported doctor-diagnosed CHD (supplementary table S2).

The CARDIoGRAMplusC4D 1000 Genomes-based GWAS (www.cardiogramplusc4d.org/data-downloads) is a meta-analysis of 48 studies, including data from 60 801 CHD cases and 123 504 controls of White European ancestry, age 42–75 years. CHD was defined as myocardial infarction, acute coronary syndrome, chronic stable angina or coronary stenosis >50% (supplementary table S2).

#### SNP selection

Based on the latest published asthma GWAS on 56 167 cases and 352 255 controls from UKB [25], we identified 116 independent SNPs associated with asthma at a p-value of 5×10^−8^. Of these 116 SNPs present in UKB, only 23 were also present in the TAGC and CARDIoGRAM datasets, but we could find a proxy (linkage disequilibrium r^2^≥0.8 with original SNP) for another 41; therefore, 64 SNPs were available for the MR analysis. All 64 SNPs had an F-statistic >10 in the GX meta-analysis of UKB and TAGC (supplementary table S3a).

#### MR analysis

In UKB, GX and GY effect estimates were obtained using logistic regression models adjusted for age, sex, centre, genotyping batch and 10 ancestry principal components. In TAGC, GX estimates were obtained from their publicly available GWAS results [26], and the same for GY estimates in CARDIoGRAM [24]. Fixed-effect meta-analyses combining UKB with TAGC and UKB with CARDIoGRAM were performed to obtain GX and GY estimates, respectively, for the main analysis.

SNP-specific estimates of the effect of asthma on CHD were obtained using the Wald estimator as the ratio of GY over GX, with both GY and GX expressed as a log odds ratio. SNP-specific MR estimates were then pooled using an inverse variance-weighted fixed effects (IVW-FE) meta-analysis.

MR results could be biased if some SNPs used as instruments for asthma were also associated with other phenotypes affecting CHD through causal pathways that are independent from asthma (horizontal pleiotropy) [27]. We investigated the presence of pleiotropic instruments by measuring heterogeneity in the SNP-specific MR results (Cochran's Q-test and I^2^-statistic) [28]. As the IVW-FE meta-analysis does not account for pleiotropy, robustness of findings to pleiotropy was investigated using several methods that control for it and are based on different assumptions about its underlying nature (details on the methods and references are provided in supplementary table S4): 1) IVW random effects (IVW-RE) meta-analysis, 2) weighted mode-based estimation, 3) weighted median estimation, 4) MR-Egger regression with SIMEX adjustment for dilution bias and 5) MR-PRESSO. Robustness to pleiotropy was also investigated by excluding SNPs identified as outliers based on their individual contributions to Cochran's Q heterogeneity [29].

All MR analyses were performed using the packages Mendelian Randomization (https://cran.r-project.org/web/packages/MendelianRandomization/index.html) and MR-PRESSO (https://github.com/rondolab/MR-PRESSO).

#### Subgroup analyses by sex (UKB alone)

In UKB, we could perform subgroup analyses investigating a possible effect modification by sex. Of the 64 SNPs used in the main analysis, some were excluded in the sex-stratified analyses because of a minor allele count <5 (four in females; five in males) or because they were weak instruments, *i.e.* F-statistic <10 (two in females; seven in males).

## Results

### Observation study

#### Patient characteristics

1 522 910 patients were included (761 455 asthma; 761 455 general population) (supplementary figure S1); mean age 35.7 years and 56.5% females. Asthma patients had a higher proportion of obesity (22.5% *versus* 17.9%), ever-smokers (63.3% *versus* 58.0%) and atopy (41.2% *versus* 19.5%) (table 1).

**TABLE 1:** Characteristics of the general population and asthma matched cohort

	**General population (n=761 455)**	**Asthma (n=761 455)**	**Total (n=1 522 910)**
**Age, years**	39.4±17.3	39.3±17.4	39.4±17.3
**Gender**			
Male	331 614 (43.6)	331 614 (43.6)	663 228 (43.6)
Female	429 841 (56.4)	429 841 (56.4)	859 682 (56.4)
**Socioeconomic status (IMD)**			
1 (least deprived)	160 915 (21.2)	152 406 (20.0)	313 321 (20.6)
2	154 087 (20.3)	150 048 (19.7)	304 135 (20.0)
3	145 773 (19.2)	145 932 (19.2)	291 705 (19.2)
4	156 527 (20.6)	159 083 (20.9)	315 610 (20.7)
5 (most deprived)	143 428 (18.9)	153 274 (20.1)	296 702 (19.5)
Missing	725 (0.1)	712 (0.1)	1437 (0.1)
**BMI category**			
Normal	221 867 (39.0)	236 544 (34.6)	458 411 (36.6)
Underweight	24 041 (4.2)	24 631 (3.6)	48 672 (3.9)
Overweight	221 491 (38.9)	268 969 (39.3)	490 460 (39.1)
Obese	102 031 (17.9)	153 966 (22.5)	255 997 (20.4)
Missing	192 025 (25.2)	77 345 (10.2)	269 370 (17.7)
**Smoking history**			
Never-smoker	285 477 (42.0)	273 009 (36.7)	558 486 (39.2)
Ex-smoker	203 833 (30.0)	258 768 (34.8)	462 601 (32.5)
Current smoker	190 382 (28.0)	212 158 (28.5)	402 540 (28.3)
Missing	81 763 (10.7)	17 520 (2.3)	99 283 (6.5)
**Atopy**			
No	613 366 (80.6)	447 710 (58.8)	1 061 076 (69.7)
Yes	148 089 (19.4)	313 745 (41.2)	461 834 (30.3)
**GP consultations**			
Quartile 1	346 642 (45.5)	161 035 (21.1)	507 677 (33.3)
Quartile 2	167 228 (22.0)	146 942 (19.3)	314 170 (20.6)
Quartile 3	141 266 (18.6)	202 850 (26.6)	344 116 (22.6)
Quartile 4	106 319 (14.0)	250 628 (32.9)	356 947 (23.4)
**OCS**			
None	761 455 (100.0)	656 384 (86.2)	1 417 839 (93.1)
1 OCS	0 (0.0)	65 615 (8.6)	65 615 (4.3)
>1 OCS	0 (0.0)	39 456 (5.2)	39 456 (2.6)
**Asthma therapy**			
No asthma	761 455 (100.0)	0 (0.0)	761 455 (50.0)
No treatment	0 (0.0)	234 405 (30.8)	234 405 (15.4)
SABA only	0 (0.0)	137 786 (18.1)	137 786 (9.0)
ICS	0 (0.0)	238 463 (31.3)	238 463 (15.7)
ICS+add-on	0 (0.0)	150 801 (19.8)	150 801 (9.9)

Data are presented as mean±sd or n (%). BMI: body mass index; IMD: Index of Multiple Deprivation; GP: general practitioner; OCS: oral corticosteroids; SABA: short-acting β_2_-agonists; ICS: inhaled corticosteroids.

#### Association between asthma and incident CHD

**FIGURE 1:**
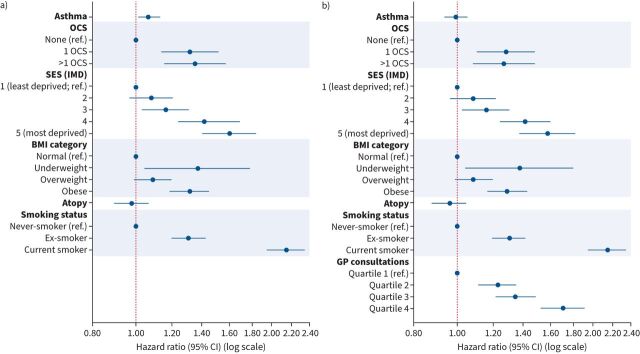
Association between asthma and incident coronary heart disease in the observational study: a) not adjusted for general practitioner (GP) consultations and b) adjusted for GP consultations. OCS: oral corticosteroids; ref.: reference; SES: socioeconomic status; IMD: Index of Multiple Deprivation; BMI: body mass index.

Asthma patients had a 6% increased risk of incident CHD in model 1 (HR 1.07, 95% CI 1.01–1.13; p<0.05) but no increase after adjusting for healthcare behaviour (model 2: HR 0.99, 95% CI 0.94–1.05; p=0.758) (figure 1, supplementary table S5 and supplementary figure S4).

#### Influence of asthma severity on the association between asthma and CHD

Asthma severity did not confound the null association between asthma and CHD (no treatment: HR 0.93, 95% CI 0.83–1.03; p=0.172; SABA only: HR 1.00, 95% CI 0.88–1.13; p=0.984; ICS: HR 1.01, 95% CI 0.93–1.10; p=0.737; ICS+add-on: HR 1.02, 95% CI 0.92–1.13; p=0.708) (supplementary table S6) or modify the association (supplementary table S7).

#### Association of GP consultation frequency with healthcare behaviour

Higher frequency of GP consultations was found for asthma, females, obesity, atopy and diabetes (supplementary table S8). Asthma and GP consultations were both associated with reporting minor health ailments. This association was much stronger for asthma than other chronic conditions (diabetes and hypertension) (supplementary figure S5). Before accounting for GP consultation frequency, asthma was significantly associated with all-cause mortality, but this effect was no longer found after accounting for this variable (supplementary figure S6). The association with minor ailments and GP consultation frequency was found even before asthma was diagnosed.

#### Factors that modified the association between asthma and CHD

Atopy, eosinophil count and smoking did not modify the association (supplementary table S9). However, females with asthma had a 11% increased risk of CHD, while males with asthma had a 9% reduced risk (supplementary figure S7).

### MR study

The sample size for our MR study was 472 411 for GX (UKB: 344 742; TAGC: 127 669) and 589 875 for GY (UKB: 405 570; CARDIoGRAM: 184 035) (supplementary table S2).

**FIGURE 2:**
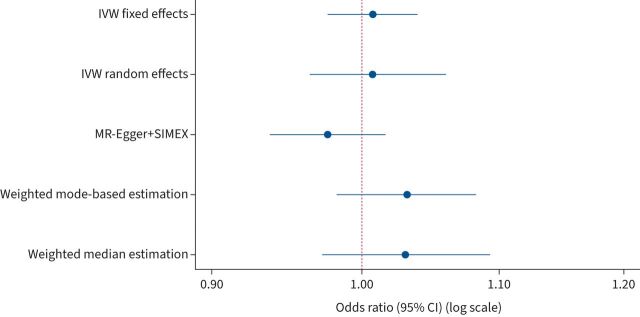
Association between asthma and coronary heart disease using different Mendelian randomisation (MR) methods. IVW: inverse variance-weighted.

We found no evidence that asthma is causally associated with CHD (IVW-FE: OR 1.01, 95% CI 0.97–1.04; p=0.676). Although we found statistical evidence of pleiotropy (p<0.001, I^2^=62%), results did not change when excluding possible pleiotropic SNPs (supplementary figure S8) and similarly null results were obtained when using methods robust to pleiotropy, including IVW-RE, MR-Egger, weighted mode-based estimation and weighted median estimation (figure 2).

Results of a sensitivity analysis performed only within UKB (one-sample MR) were consistent with the main analysis (supplementary table S9).

In the MR analysis stratified by sex (data available for UKB only), we did not find evidence of a causal association between asthma and CHD in females or males (IVW-FE: females: OR 1.02, 95% CI 0.97–1.06; p=0.569; males: OR 1.00, 95% CI 0.96–1.05; p=0.877) (supplementary table S10). Further analyses with pleiotropy-robust methods found similar results.

## Discussion

In our observational analysis, without accounting for healthcare behaviour, we found a 7% increased risk of incident CHD associated with asthma, comparable to previous observational studies [18]. However, after accounting for healthcare behaviour, we found no increased risk, suggesting previous studies may have suffered from detection bias. Furthermore, in our MR analysis, we found no evidence of a causal association between asthma and CHD.

Observational studies cannot easily distinguish a causal from a spurious association due to possible residual confounding and reverse causation; therefore, we also used MR, a method where genetic variants are used as instrumental variables for the exposure (asthma), which is not affected by these issues. However, the validity of MR relies on certain instrumental variable assumptions, the most important being the absence of pleiotropy. Despite some evidence of pleiotropy for a few SNPs, our results remained consistent when we applied methods robust to pleiotropy and when we excluded possible pleiotropic SNPs, suggesting that pleiotropy did not bias our results.

We also investigated several factors that could influence the association between asthma and CHD. Asthma severity and indicators of type 2 inflammation were not found to confound or modify the association. However, use of OCS for an asthma exacerbation did increase the risk of CHD. OCS increase cardiovascular risk through enhancement of metabolic syndrome and mineralocorticoid effects; even low doses increase CHD risk in asthma and inflammatory diseases [30, 31].

Multiple earlier studies found sex to be a modifier of the association between asthma and CHD, with several reporting that only females carried a higher risk [18]. Our observational study also found females were at increased risk, but additionally that males with asthma appeared to be protected compared with males without asthma. This discrepancy is difficult to explain biologically and our MR analysis, which does not suffer from classical confounding or detection bias, found no difference by sex. It is also notable that females and males had substantial differences in their healthcare behaviour, suggesting possible detection bias. Another proposed modifier was smoking (although findings were inconsistent across earlier studies); here again we found no evidence of modification.

Asthma patients were more likely to visit their GP practice for minor ailments than the general population, even compared with patients with other chronic conditions (diabetes and hypertension). This healthcare-seeking behaviour may be due to the high prevalence of anxiety associated with asthma [32], general preponderance towards healthcare-seeking behaviour, as seen with coronavirus disease 2019 [21], or unknown factors. The GP consultations quartile with the highest consultations was strongly associated with all-cause mortality, suggesting a potential proxy for frailty. Notably, not accounting for this led to the finding that asthma patients had increased all-cause mortality, which we interpret as biased as mortality rates directly linked to asthma are very low. Patients that exhibit more healthcare-seeking behaviour are more likely to report symptoms, including milder symptoms, increasing the risk of detection bias.

A key strength of our study is the triangulation approach, where the use of both traditional observational and MR analyses, based on methods that suffer from different limitations, allows us to broach causation. Additionally, our observational analysis tried to account for detection bias, included a large sample size and did not suffer from selection or recall bias. However, such data can suffer from misclassification, *e.g.* incorrectly diagnosing people with COPD as asthma; however, if this was true for many people, we would have observed an association with CHD, as COPD is a known risk factor. Similarly, our MR analysis is based on genetic data from large sample sizes and its results appeared robust to pleiotropy, although this can never be completely ruled out. Our approach was only partially a two-sample MR, with UKB contributing to both GX and GY estimates. However, bias away from the null resulting from sample overlapping [33] is unlikely as we excluded weak instruments and, furthermore, we did not find evidence of a causal effect. All our MR analyses were restricted to White European participants; therefore, we cannot extrapolate our findings to different ethnicities.

We conclude that asthma, regardless of phenotype or sex, is not a risk factor for CHD. Our findings contrast with previous observational studies which may have suffered from detection bias and residual confounding.

## Supplementary material

10.1183/13993003.01788-2023.Supp1 **Please note:** supplementary material is not edited by the Editorial Office, and is uploaded as it has been supplied by the author.
Supplementary material ERJ-01788-2023.SUPPLEMENT

## Shareable PDF

10.1183/13993003.01788-2023.Shareable1 This one-page PDF can be shared freely online.
Shareable PDF ERJ-01788-2023.Shareable
