# Supplementary material for: Asthma and incident coronary heart disease: an observational and Mendelian randomisation study
Source: Eur Respir J. 2023 Nov 30;62(5):2301788. doi: 10.1183/13993003.01788-2023 (PMC10695770; doi:10.1183/13993003.01788-2023)

Table S1. List of Read codes for asthma and ICD-10 codes for coronary heart disease

Read Code	Description
173A.00	Exercise induced asthma
173c.00	Occupational asthma
173d.00	Work aggravated asthma
1780	Aspirin induced asthma
1781	Asthma trigger - pollen
1782	Asthma trigger - tobacco smoke
1783	Asthma trigger - warm air
1784	Asthma trigger - emotion
1785	Asthma trigger - damp
1786	Asthma trigger - animals
1787	Asthma trigger - seasonal
1788	Asthma trigger - cold air
1789	Asthma trigger - respiratory infection
178A.00	Asthma trigger - airborne dust
178B.00	Asthma trigger - exercise
1O2..00	Asthma confirmed
388t.00	Royal College of Physicians asthma assessment
388t000	Royal College Physician asthma assessment 3 question score
38DL.00	Asthma control test
38DT.00	Asthma control questionnaire
38DV.00	Mini asthma quality of life questionnaire
661M100	Asthma self-management plan agreed
661N100	Asthma self-management plan review
663..11	Asthma monitoring
663N.00	Asthma disturbing sleep
663N000	Asthma causing night waking
663N100	Asthma disturbs sleep weekly
663N200	Asthma disturbs sleep frequently
663O000	Asthma never disturbs sleep
663P.00	Asthma limiting activities
663P000	Asthma limits activities 1 to 2 times per month
663P100	Asthma limits activities 1 to 2 times per week
663P200	Asthma limits activities most days
663U.00	Asthma management plan given
663V.00	Asthma severity
663V000	Occasional asthma
663V100	Mild asthma
663V200	Moderate asthma
663V300	Severe asthma
663d.00	Emergency asthma admission since last appointment
663e.00	Asthma restricts exercise
663	Asthma sometimes restricts exercise
6.63E+102	Asthma severely restricts exercise

|

663f.00	Asthma never restricts exercise
663j.00	Asthma - currently active
663m.00	Asthma accident and emergency attendance since last visit
663n.00	Asthma treatment compliance satisfactory
663p.00	Asthma treatment compliance unsatisfactory
663q.00	Asthma daytime symptoms
663r.00	Asthma causes night symptoms 1 to 2 times per month
663s.00	Asthma never causes daytime symptoms
663t.00	Asthma causes daytime symptoms 1 to 2 times per month
663u.00	Asthma causes daytime symptoms 1 to 2 times per week
663v.00	Asthma causes daytime symptoms most days
663w.00	Asthma limits walking up hills or stairs
663x.00	Asthma limits walking on the flat
663y.00	Number of asthma exacerbations in past year
66Y5.00	Change in asthma management plan
66Y9.00	Step up change in asthma management plan
66YA.00	Step down change in asthma management plan
66YC.00	Absent from work or school due to asthma
66YJ.00	Asthma annual review
66YK.00	Asthma follow-up
66YP.00	Asthma night-time symptoms
66YQ.00	Asthma monitoring by nurse
66YR.00	Asthma monitoring by doctor
66Yp.00	Asthma review using Roy College of Physicians three questions
66Yq.00	Asthma causes night-time symptoms 1 to 2 times per week
66Yr.00	Asthma causes symptoms most nights
66Ys.00	Asthma never causes night symptoms
66Yu.00	Number days absent from school due to asthma in past 6 month
679J000	Health education - asthma self-management
679J100	Health education - structured asthma discussion
679J200	Health education - structured patient focused asthma discuss
8791	Further asthma - drug prevent.
8794	Asthma control step 1
8795	Asthma control step 2
8796	Asthma control step 3
8797	Asthma control step 4
8798	Asthma control step 5
8B3j.00	Asthma medication review
8CMA000	Patient has a written asthma personal action plan
8CR0.00	Asthma clinical management plan
8H2P.00	Emergency admission
9NNX.00	Under care of asthma specialist nurse
9OJ1.00	Attends asthma monitoring
9OJA.00	Asthma monitoring check done
9OJA.11	Asthma monitored

|

H312000	Chronic asthmatic bronchitis
H33..00	Asthma
H33..11	Bronchial asthma
H330.00	Extrinsic (atopic) asthma
H330.11	Allergic asthma
H330.13	Hay fever with asthma
H330.14	Pollen asthma
H330000	Extrinsic asthma without status asthmaticus
H330011	Hay fever with asthma
H330100	Extrinsic asthma with status asthmaticus
H330111	Extrinsic asthma with asthma attack
H330z00	Extrinsic asthma NOS
H331.00	Intrinsic asthma
H331.11	Late onset asthma
H331000	Intrinsic asthma without status asthmaticus
H331100	Intrinsic asthma with status asthmaticus
H331111	Intrinsic asthma with asthma attack
H331z00	Intrinsic asthma NOS
H333.00	Acute exacerbation of asthma
H334.00	Brittle asthma
H335.00	Chronic asthma with fixed airflow obstruction
H33z.00	Asthma unspecified
H33z000	Status asthmaticus NOS
H33z011	Severe asthma attack
H33z100	Asthma attack
H33z111	Asthma attack NOS
H33z200	Late-onset asthma
H33zz00	Asthma NOS
H33zz11	Exercise induced asthma
H33zz12	Allergic asthma NEC

Table S2. Selected samples, exposures and outcomes for the genetic instruments used in the MR study

GX/GY	Study	Cases	Controls	Total	Definition	Reference
GX (Asthma)	UKB	**Original sample**			Data-field 22127 - Self-reported doctor-diagnosed asthma	• J Allergy Clin Immunol. 146(2):327-329 (2020)<br>• Nature.2018;562(7726):203-209. doi:10.1038/s41586-018-0579-z
		65,677	422,700	488,377		
		**Sample included**				
		49,008	295,734	344,742		
GX (Asthma)	TAGC	**Original sample**			Doctor's diagnosis and/or standardised questionnaire, depending on the study.	• Nat Genet. 2018;50(1):42-53. doi:10.1038/s41588-017-0014-7
		23,948	118,538	142,486		
		**Sample included**				
		19,954	107,715	127,669		
GY (CHD)	UKB	**Original sample**			Data-field 20002 (1075) - Self-reported heart attack (myocardial infarction) or angina<br>Data-field 20002 (1074) - Self-reported angina<br>Data-field 6150 (1) - Doctor diagnosed heart attack<br>Data-field 6150 (2) - Doctor diagnosed angina<br>Data-field 3894 - Age heart attack diagnosed<br>Data-field 20004 (1070) - Coronary angioplasty (PTCA) +/- stent<br>Data-field 20004 (1095) - Coronary artery bypass grafts (cabg)<br>Data-field 20004 (1523) - Triple heart bypass	• Nature. 2018;562(7726):203-209. doi:10.1038/s41586-018-0579-z
		37,476		488,377		
		**Sample included**				
		19,294	386,276	405,570		
GY (CHD)	CARDIoGRAM	**Original sample**			Depending on specific study.<br>KPC: ICD 9 410./41.3/X.<br>Academic Medical Center Amsterdam Premature: Symptomatic CAD<br>KORA F3 study: CAD/MI based on coronary angiogram<br>Cardiogenics: MI within last 3-36 months.<br>METSIM: CAD (angiography confirmed)<br>DILGOM: CAD<br>DUKE: CAD<br>EGCUT: CAD/MI (ICD10 I20-I26)<br>EPIC: CAD/MI: (ICD9 410-414 or ICD10 I20-I25)<br>FGENTCARD: CAD/MI<br>FRISCII: CAD/MI<br>GLACIER: Control population<br>GODARTS: CAD/MI<br>HPS: CAD/MI<br>ITH: CAD/MI within 24 hours of symptom onset<br>LOLIPOP: CAD/MI (CABG/PCI)<br>LURIC-EMIL: Angiographically confirmed CAD<br>METSIM: Angiographically confirmed CAD, MI, balloon angioplasty, coronary bypass<br>MORGAM: CAD. Revascularisation<br>OHGS: CAD/MI<br>PIVUS: ICD-8 and ICD-9 code 410, ICD-10 codes I21-I22<br>PMB: CAD/MI<br>PopGen: CAD (at least 70% stenosis in a major coronary)<br>PROCARDISS: Symptomatic CAD before age 66<br>PROMIS Study. CAD/MI within the previous 24 hours<br>SCARF-SHEEP: First confirmed myocardial infarction<br>STR: (ICD-8 and ICD-9 code 410, ICD-10 codes I21-I22) or unstable angina (ICD-8 code 411, ICD-9 code 411B, ICD-10 code I20.0).<br>THISEAS study: First-ever mi before age of 70 yrs; first CAD<br>Uppsala Longitudinal Study of Adult men: (ICD-8 and ICD-9 code 410, ICD-10 codes I21-I22) or unstable angina (ICD-8 code 411, ICD-9 code 411B, ICD-10 code I20.0)	• Nat Genet. 2015;47(10):1121-1130. doi:10.1038/ng.3396
		63,746	130,681	194,427		
		**Sample included**				
		60,801	123,504	184,035		

* the data fields for UKB are linked with an 'or'.

Table S3 A. Genetic instruments from UKB and TAGC used in the main analysis

N	SNP	Proxy SNP	r2	Chr	Position	EA	EAF	F	Beta GX	SE GX	P-value	Beta GY	SE GY	P-value
1	rs10178845	–	1	2	8443803	a	0.23	36.52	0.042	0.007	1.86E-09	0.009	0.014	4.9E-01
2	rs10795633	rs7091181	1	10	8513624	c	0.39	49.37	0.053	0.008	1.74E-12	-0.002	0.008	7.7E-01
3	rs10912564	–	1	1	1.73E+08	c	0.62	18.90	0.030	0.007	1.51E-05	0.002	0.007	8.1E-01
4	rs11042902	–	1	11	10655623	c	0.83	16.93	0.029	0.007	4.11E-05	0.001	0.007	8.7E-01
5	rs11088309	rs17812893	1	21	36437725	c	0.06	54.59	0.076	0.010	1.31E-13	0.002	0.008	7.7E-01
6	rs11178649	rs3942254	1	12	71455871	t	0.25	38.81	0.046	0.007	4.91E-10	0.000	0.015	9.9E-01
7	rs112267124	rs17581728	0.9	17	73830996	g	0.84	25.95	0.043	0.009	3.93E-07	-0.027	0.008	8.9E-04
8	rs112502960	rs12940887	1	17	47402807	g	0.82	78.65	0.067	0.008	4.69E-19	-0.003	0.011	7.7E-01
9	rs11256026	–	1	10	9054689	a	0.55	11.89	0.023	0.007	6.05E-04	0.012	0.008	1.6E-01
10	rs113981909	rs7069034	1	10	8321068	a	0.06	32.53	0.067	0.012	1.28E-08	0.015	0.010	1.6E-01
11	rs12365699	–	1	11	1.19E+08	a	0.07	17.49	0.037	0.009	2.71E-05	0.023	0.009	9.8E-03
12	rs12368672	rs167769	0.9	12	57503775	c	0.29	117.46	0.080	0.007	1.97E-27	-0.003	0.008	6.7E-01
13	rs12470864	rs950880	1	2	1.03E+08	g	0.70	213.40	0.108	0.007	5.06E-48	-0.008	0.008	3.4E-01
14	rs13099273	rs12639588	0.9	3	1.88E+08	t	0.36	68.40	0.061	0.007	1.10E-16	0.001	0.007	8.9E-01
15	rs1444782	rs962993	1	10	9053132	a	0.27	227.85	0.112	0.007	8.46E-52	0.008	0.008	3.4E-01
16	rs1689510	rs772921	1	12	56403577	g	0.20	91.50	0.073	0.008	1.68E-21	-0.003	0.007	6.6E-01
17	rs1789358	rs568038	1	11	1.11E+08	g	0.37	50.57	0.058	0.008	8.23E-13	0.002	0.007	7.9E-01
18	rs1800797	–	1	7	22766221	a	0.14	11.01	0.022	0.007	8.83E-04	0.006	0.008	4.1E-01
19	rs1837253	–	1	5	1.1E+08	t	0.38	91.39	0.072	0.008	6.77E-22	0.011	0.008	2.1E-01
20	rs1870140	rs6586030	0.9	10	82254047	g	0.93	20.07	0.045	0.010	7.83E-06	-0.010	0.007	1.9E-01
21	rs2025758	–	1	10	8841669	c	0.38	37.12	0.040	0.007	1.07E-09	0.004	0.008	6.4E-01
22	rs2255088	–	1	10	6625378	t	0.59	23.73	0.034	0.007	1.27E-06	-0.005	0.007	5.5E-01
23	rs2296618	–	1	1	1.99E+08	g	0.22	28.70	0.053	0.010	8.11E-08	0.030	0.010	3.9E-03
24	rs2596544	rs1611400	0.9	6	29890918	t	0.19	94.78	0.085	0.009	1.94E-22	0.035	0.016	3.3E-02
25	rs2995089	–	1	1	1.67E+08	a	0.16	40.50	0.042	0.007	1.89E-10	0.004	0.007	5.7E-01
26	rs3024971	–	1	12	57493727	g	0.07	76.60	0.095	0.011	1.46E-18	-0.013	0.011	2.5E-01
27	rs3129777	rs2187668	0.8	6	32605884	c	0.09	191.81	0.129	0.009	2.16E-43	0.018	0.010	9.2E-02
28	rs343478	rs343488	0.9	9	6064071	a	0.50	58.50	0.057	0.007	1.57E-14	-0.015	0.010	1.4E-01
29	rs35225972	rs11245962	1	11	1111164	g	0.50	36.00	0.047	0.008	1.55E-09	0.002	0.014	9.0E-01
30	rs35441874	rs12935657	1	16	11219041	a	0.15	149.41	0.104	0.009	4.64E-34	0.005	0.007	5.1E-01
31	rs35570272	rs9828592	0.7	3	33044339	g	0.63	64.22	0.059	0.007	1.68E-15	-0.001	0.014	9.2E-01
32	rs35621564	rs12700215	0.9	7	20584559	g	0.31	58.44	0.058	0.008	2.47E-14	-0.022	0.007	2.3E-03
33	rs3749833	rs1023518	1	5	1.32E+08	t	0.27	112.83	0.087	0.008	1.57E-26	-0.008	0.013	5.2E-01
34	rs3785356	rs4787948	1	16	27341059	c	0.26	71.93	0.067	0.008	3.17E-17	0.001	0.007	8.9E-01
35	rs3827780	rs2614257	1	6	1.36E+08	a	0.61	26.95	0.038	0.007	2.14E-07	0.001	0.008	8.7E-01
36	rs4247364	rs4248914	0.8	17	43336480	c	0.30	48.29	0.055	0.008	3.39E-12	0.020	0.008	9.7E-03
37	rs4480384	–	1	1	8659714	a	0.37	26.06	0.034	0.007	3.10E-07	-0.015	0.008	4.5E-02
38	rs4749785	–	1	10	8603844	c	0.75	24.72	0.036	0.007	6.16E-07	0.007	0.008	3.8E-01
39	rs4795401	rs11557467	1	17	38028634	g	0.35	253.38	0.116	0.007	1.31E-57	-0.003	0.007	7.0E-01
40	rs479844	–	1	11	65551957	a	0.64	19.91	0.029	0.007	7.58E-06	0.002	0.010	8.2E-01
41	rs56328339	rs17607589	0.9	3	1.88E+08	t	0.06	44.09	0.066	0.010	2.76E-11	-0.005	0.008	5.3E-01
42	rs5743618	rs12233670	0.9	4	38787216	a	0.80	115.81	0.095	0.009	5.03E-27	0.008	0.010	3.8E-01
43	rs6457541	rs9268005	1	6	32224388	g	0.29	23.59	0.037	0.008	1.10E-06	-0.008	0.008	2.8E-01
44	rs6594499	–	1	5	1.1E+08	a	0.65	64.50	0.051	0.006	1.56E-15	-0.016	0.012	1.8E-01
45	rs6687430	–	1	1	10633245	g	0.35	18.73	0.028	0.006	1.61E-05	0.055	0.008	5.5E-13
46	rs7219483	rs1048572	1	17	38797242	a	0.28	36.15	0.048	0.008	1.86E-09	0.014	0.013	2.8E-01
47	rs72823641	rs11690644	1	2	1.03E+08	a	0.06	313.52	0.193	0.011	1.35E-70	0.009	0.008	2.6E-01

48	rs72833417	rs10514934	1	17	45812124	a	0.08	38.24	0.067	0.011	6.81E-10	-0.006	0.013	6.6E-01
49	rs7423358	–	1	2	2.29E+08	t	0.34	11.98	0.026	0.008	0.0005475	0.001	0.011	9.2E-01
50	rs7626218	rs1201296	0.7	3	1.77E+08	t	0.46	43.91	0.050	0.008	2.67E-11	-0.002	0.008	7.6E-01
51	rs76493820	rs11746314	0.9	5	1.57E+08	c	0.02	31.70	0.087	0.015	1.78E-08	-0.010	0.008	2.3E-01
52	rs770379212	rs7671357	0.7	4	1.23E+08	ca	0.71	72.03	0.066	0.008	1.43E-17	-0.010	0.008	1.8E-01
53	rs770792291	rs17105278	1	14	68728479	aat	0.31	55.05	0.060	0.008	1.51E-13	-0.015	0.007	3.8E-02
54	rs7735519	–	1	5	1.1E+08	c	0.67	46.33	0.060	0.009	1.17E-11	-0.003	0.007	6.4E-01
55	rs7770794	–	1	6	1.55E+08	g	0.79	24.02	0.035	0.007	1.03E-06	0.004	0.007	6.2E-01
56	rs7936312	rs2155219	0.9	11	76299194	g	0.56	246.02	0.115	0.007	5.81E-56	0.014	0.009	1.0E-01
57	rs7961712	–	1	12	94604963	g	0.11	19.22	0.040	0.009	1.17E-05	-0.013	0.012	2.6E-01
58	rs802731	rs802734	0.9	6	1.28E+08	c	0.18	56.53	0.061	0.008	6.59E-14	0.012	0.008	1.5E-01
59	rs802731	rs813915	0.7	6	1.28E+08	c	0.18	56.53	0.061	0.008	6.59E-14	-0.006	0.007	4.4E-01
60	rs848	–	1	5	1.32E+08	c	0.62	48.32	0.057	0.008	3.26E-12	-0.030	0.008	1.4E-04
61	rs919826	rs1107677	1	9	23587027	c	0.38	30.48	0.040	0.007	3.34E-08	0.006	0.007	4.5E-01
62	rs992969	–	1	9	6209697	g	0.78	99.18	0.073	0.007	1.26E-23	-0.010	0.012	4.1E-01

|

Table S3 B. Genetic instruments from UKB used in the sex-stratified analyses (male)

N	SNP	Chr	Position	EA	EAF	F	Beta GX	SE GX	P-value	Beta GY	SE GY	P-value
1	rs2844581	6	31332703	C	0.10	10.16	0.040	0.013	1.44E-03	-0.022	1.54E-02	1.59E-01
2	rs17812893	21	36437725	G	0.06	11.45	0.051	0.015	7.16E-04	0.006	1.84E-02	7.38E-01
3	rs2614257	6	135676404	C	0.32	11.46	0.036	0.011	7.13E-04	0.005	1.30E-02	6.77E-01
4	rs17581728	17	73830996	T	0.16	11.65	0.043	0.013	6.41E-04	-0.033	1.52E-02	2.88E-02
5	rs1800797	7	22766221	G	0.86	12.09	0.038	0.011	5.07E-04	-0.013	1.31E-02	3.07E-01
6	rs4248914	17	43336480	C	0.23	15.65	0.048	0.012	7.61E-05	-0.003	1.48E-02	8.44E-01
7	rs1107677	9	23587027	C	0.61	16.53	0.044	0.011	4.79E-05	-0.013	1.29E-02	3.01E-01
8	rs7961712	12	94604963	A	0.89	16.80	0.062	0.015	4.16E-05	0.022	1.80E-02	2.25E-01
9	rs7069034	10	8321068	G	0.94	17.98	0.073	0.017	2.23E-05	0.014	2.04E-02	4.77E-01
10	rs343488	9	6064071	C	0.50	19.40	0.048	0.011	1.06E-05	0.000	1.30E-02	9.76E-01
11	rs2255088	10	6625378	C	0.41	20.16	0.051	0.011	7.11E-06	-0.019	1.37E-02	1.62E-01
12	rs11042902	11	10655623	T	0.17	20.79	0.053	0.012	5.12E-06	-0.023	1.40E-02	9.67E-02
13	rs1048572	17	38797242	G	0.74	21.27	0.055	0.012	3.99E-06	-0.007	1.42E-02	6.37E-01
14	rs3942254	12	71455871	G	0.75	21.29	0.050	0.011	3.95E-06	-0.010	1.31E-02	4.38E-01
15	rs12365699	11	118743286	G	0.93	22.78	0.070	0.015	1.82E-06	-0.010	1.73E-02	5.50E-01
16	rs6687430	1	10633245	A	0.52	23.79	0.053	0.011	1.07E-06	0.012	1.29E-02	3.74E-01
17	rs568038	11	111432161	T	0.64	24.34	0.058	0.012	8.08E-07	-0.022	1.42E-02	1.28E-01
18	rs10912564	1	173170618	T	0.38	25.91	0.059	0.012	3.58E-07	-0.006	1.40E-02	6.74E-01
19	rs2296618	1	198666232	A	0.78	27.69	0.085	0.016	1.42E-07	-0.020	1.91E-02	2.98E-01
20	rs7671357	4	123126450	A	0.29	27.85	0.060	0.011	1.31E-07	0.003	1.37E-02	8.20E-01
21	rs11245962	11	1111164	T	0.50	28.63	0.062	0.012	8.77E-08	-0.012	1.38E-02	3.73E-01
22	rs4787948	16	27341059	G	0.35	28.80	0.063	0.012	8.04E-08	-0.026	1.41E-02	6.51E-02
23	rs2187668	6	32605884	T	0.01	28.89	0.079	0.015	7.66E-08	-0.017	1.79E-02	3.50E-01
24	rs4749785	10	8603844	T	0.25	30.31	0.066	0.012	3.69E-08	-0.022	1.45E-02	1.36E-01
25	rs2995089	1	167431193	G	0.84	30.92	0.061	0.011	2.69E-08	-0.010	1.31E-02	4.33E-01
26	rs802734	6	128278798	G	0.19	31.49	0.064	0.011	2.01E-08	0.001	1.39E-02	9.70E-01
27	rs479844	11	65551957	G	0.36	32.79	0.062	0.011	1.03E-08	0.009	1.30E-02	4.82E-01
28	rs4480384	1	8659714	G	0.63	33.43	0.065	0.011	7.38E-09	0.031	1.35E-02	2.28E-02
29	rs2025758	10	8841669	T	0.62	33.77	0.063	0.011	6.20E-09	0.006	1.30E-02	6.26E-01
30	rs9828592	3	33044339	C	0.39	34.80	0.064	0.011	3.66E-09	0.006	1.30E-02	6.50E-01
31	rs17105278	14	68728479	C	0.31	35.20	0.071	0.012	2.97E-09	-0.004	1.44E-02	8.00E-01
32	rs12700215	7	20584559	G	0.68	38.69	0.070	0.011	4.97E-10	-0.016	1.35E-02	2.25E-01
33	rs813915	6	128287313	T	0.24	38.82	0.077	0.012	4.66E-10	0.010	1.51E-02	5.01E-01
34	rs12233670	4	38787216	C	0.60	46.58	0.092	0.014	8.79E-12	-0.006	1.62E-02	7.08E-01
35	rs2596560	6	31355318	C	0.24	46.70	0.084	0.012	8.27E-12	-0.005	1.50E-02	7.40E-01
36	rs12940887	17	47402807	T	0.19	48.34	0.077	0.011	3.59E-12	0.074	1.33E-02	2.45E-08
37	rs7735519	5	110153341	A	0.33	49.08	0.105	0.015	2.46E-12	-0.010	1.83E-02	5.82E-01
38	rs772921	12	56403577	T	0.20	50.66	0.080	0.011	1.10E-12	0.017	1.36E-02	2.08E-01
39	rs10178845	2	8443803	G	0.77	54.38	0.087	0.012	1.65E-13	-0.011	1.41E-02	4.40E-01
40	rs167769	12	57503775	T	0.27	57.31	0.082	0.011	3.71E-14	0.009	1.31E-02	4.85E-01
41	rs3024971	12	57493727	T	0.07	61.40	0.139	0.018	4.66E-15	0.014	2.08E-02	5.06E-01
42	rs848	5	131996500	A	0.38	64.43	0.110	0.014	9.99E-16	0.022	1.67E-02	1.86E-01
43	rs1023518	5	131793772	T	0.31	71.66	0.102	0.012	2.56E-17	0.022	1.46E-02	1.24E-01
44	rs12935657	16	11219041	G	0.86	82.49	0.114	0.013	1.06E-19	-0.018	1.50E-02	2.44E-01
45	rs962993	10	9053132	C	0.75	97.19	0.107	0.011	6.29E-23	0.019	1.30E-02	1.50E-01
46	rs6594499	5	110470137	C	0.35	104.90	0.110	0.011	1.29E-24	0.011	1.29E-02	3.89E-01
47	rs11557467	17	38028634	G	0.62	136.77	0.125	0.011	1.35E-31	0.003	1.29E-02	8.42E-01

48	rs2155219	11	76299194	T	0.54	142.88	0.128	0.011	6.24E-33	-0.004	1.29E-02	7.82E-01
49	rs1837253	5	110401872	C	0.62	154.36	0.154	0.012	1.93E-35	0.008	0.014723	5.80E-01
50	rs950880	2	102932562	A	0.30	154.89	0.136	0.011	1.48E-35	-0.022	1.32E-02	1.00E-01
51	rs11690644	2	102914214	A	0.95	188.31	0.225	0.016	7.44E-43	0.005	1.90E-02	7.75E-01
52	rs992969	9	6209697	A	0.22	194.51	0.169	0.012	3.29E-44	-0.006	1.48E-02	6.99E-01

|
Table S3 C. Genetic instruments from UKB used in the sex-stratified analyses (female)

N	SNP	Chr	Position	EA	EAF	F	Beta GX	SE GX	P-value	Beta GY	SE GY	P-value
1	rs6586030	10	82254047	A	0.07	10.52	0.042	0.013	1.18E-03	-0.014	0.027	6.09E-01
2	rs7069034	10	8321068	G	0.94	12.38	0.052	0.015	4.33E-04	0.050	0.031	1.10E-01
3	rs12365699	11	118743286	G	0.93	12.77	0.045	0.013	3.53E-04	0.010	0.027	7.19E-01
4	rs2614257	6	135676404	C	0.32	13.39	0.034	0.009	2.54E-04	0.007	0.020	7.12E-01
5	rs11245962	11	1111164	T	0.50	13.65	0.037	0.010	2.20E-04	-0.002	0.021	9.27E-01
6	rs6687430	1	10633245	A	0.51	13.79	0.035	0.009	2.04E-04	-0.013	0.020	4.98E-01
7	rs11042902	11	10655623	T	0.17	14.53	0.038	0.010	1.38E-04	0.020	0.022	3.53E-01
8	rs17581728	17	73830996	T	0.16	14.71	0.042	0.011	1.25E-04	0.029	0.023	2.15E-01
9	rs1107677	9	23587027	C	0.61	15.19	0.036	0.009	9.72E-05	-0.003	0.020	8.65E-01
10	rs1048572	17	38797242	G	0.74	16.18	0.041	0.010	5.75E-05	-0.017	0.022	4.36E-01
11	rs11256026	10	9054689	G	0.45	16.55	0.039	0.010	4.75E-05	-0.013	0.021	5.19E-01
12	rs11746314	5	156752957	G	0.02	16.66	0.080	0.020	4.47E-05	-0.052	0.043	2.21E-01
13	rs3942254	12	71455871	G	0.75	18.55	0.041	0.009	1.66E-05	0.017	0.020	3.99E-01
14	rs813915	6	128287313	T	0.24	18.68	0.047	0.011	1.55E-05	0.025	0.023	2.88E-01
15	rs1800797	7	22766221	G	0.86	18.98	0.041	0.009	1.32E-05	-0.041	0.020	4.32E-02
16	rs4749785	10	8603844	T	0.25	19.91	0.047	0.010	8.13E-06	-0.022	0.022	3.21E-01
17	rs802734	6	128278798	G	0.19	20.18	0.045	0.010	7.05E-06	0.033	0.021	1.19E-01
18	rs10912564	1	173170618	T	0.38	20.35	0.045	0.010	6.44E-06	-0.016	0.022	4.57E-01
19	rs479844	11	65551957	G	0.36	20.66	0.042	0.009	5.50E-06	0.027	0.020	1.67E-01
20	rs2187668	6	32605884	T	0.01	21.36	0.059	0.013	3.82E-06	-0.030	0.027	2.74E-01
21	rs2296618	1	198666232	A	0.78	22.40	0.065	0.014	2.22E-06	-0.001	0.029	9.81E-01
22	rs7770794	6	154627823	A	0.21	22.52	0.049	0.010	2.08E-06	-0.014	0.022	5.20E-01
23	rs4480384	1	8659714	G	0.63	23.34	0.047	0.010	1.36E-06	0.005	0.021	8.21E-01
24	rs7961712	12	94604963	A	0.89	24.17	0.065	0.013	8.81E-07	0.054	0.028	5.28E-02
25	rs568038	11	111432161	T	0.65	24.65	0.051	0.010	6.89E-07	-0.026	0.022	2.30E-01
26	rs4248914	17	43336480	C	0.23	25.26	0.053	0.011	5.02E-07	0.011	0.023	6.18E-01
27	rs12700215	7	20584559	G	0.68	25.74	0.049	0.010	3.91E-07	-0.030	0.021	1.50E-01
28	rs17105278	14	68728479	C	0.31	25.80	0.053	0.010	3.79E-07	0.039	0.022	7.62E-02
29	rs10514934	17	45812124	C	0.07	27.61	0.071	0.014	1.48E-07	0.003	0.029	9.14E-01
30	rs9268005	6	32224388	A	0.72	29.16	0.053	0.010	6.65E-08	0.000	0.021	9.82E-01
31	rs9828592	3	33044339	C	0.39	29.48	0.051	0.009	5.66E-08	-0.021	0.020	2.92E-01
32	rs2255088	10	6625378	C	0.41	30.14	0.054	0.010	4.02E-08	-0.023	0.021	2.81E-01
33	rs4787948	16	27341059	G	0.35	37.13	0.062	0.010	1.11E-09	-0.018	0.022	4.11E-01
34	rs10178845	2	8443803	G	0.77	38.12	0.063	0.010	6.65E-10	0.019	0.022	3.75E-01
35	rs7091181	10	8513624	G	0.64	39.51	0.060	0.010	3.26E-10	0.015	0.021	4.63E-01
36	rs12940887	17	47402807	T	0.19	39.67	0.061	0.010	3.01E-10	0.060	0.020	3.58E-03
37	rs7671357	4	123126450	A	0.29	43.46	0.065	0.010	4.32E-11	0.012	0.021	5.71E-01
38	rs2596560	6	31355318	C	0.24	43.53	0.071	0.011	4.18E-11	-0.031	0.023	1.82E-01
39	rs343488	9	6064071	C	0.50	45.32	0.063	0.009	1.67E-11	0.026	0.020	1.89E-01
40	rs2025758	10	8841669	T	0.62	45.65	0.063	0.009	1.41E-11	-0.010	0.020	6.10E-01
41	rs17812893	21	36437725	G	0.06	46.62	0.089	0.013	8.62E-12	-0.011	0.028	7.06E-01
42	rs772921	12	56403577	T	0.20	46.93	0.067	0.010	7.36E-12	0.016	0.021	4.37E-01
43	rs2995089	1	167431193	G	0.84	49.33	0.067	0.010	2.16E-12	-0.009	0.020	6.67E-01
44	rs1023518	5	131793772	T	0.31	52.29	0.076	0.010	4.79E-13	0.022	0.023	3.21E-01
45	rs12233670	4	38787216	C	0.59	59.76	0.091	0.012	1.07E-14	0.028	0.025	2.67E-01
46	rs7735519	5	110153341	A	0.33	66.96	0.107	0.013	2.78E-16	0.013	0.028	6.46E-01
47	rs167769	12	57503775	T	0.27	68.64	0.078	0.009	1.18E-16	-0.010	0.020	6.22E-01

48	rs6594499	5	110470137	C	0.35	71.24	0.079	0.009	3.16E-17	0.003	0.020	8.84E-01
49	rs848	5	131996500	A	0.38	75.12	0.103	0.012	4.42E-18	0.062	0.025	1.38E-02
50	rs12935657	16	11219041	G	0.86	77.84	0.096	0.011	1.11E-18	0.016	0.023	4.90E-01
51	rs950880	2	102932562	A	0.30	84.99	0.088	0.009	2.99E-20	0.019	0.020	3.56E-01
52	rs3024971	12	57493727	T	0.93	94.42	0.150	0.015	2.56E-22	-0.030	0.032	3.42E-01
53	rs1837253	5	110401872	C	0.62	102.69	0.108	0.011	3.92E-24	-0.034	0.023	1.33E-01
54	rs2155219	11	76299194	T	0.54	103.50	0.094	0.009	2.60E-24	-0.014	0.020	4.85E-01
55	rs11557467	17	38028634	G	0.62	117.32	0.101	0.009	2.45E-27	0.016	0.020	4.05E-01
56	rs992969	9	6209697	A	0.22	123.36	0.118	0.011	1.16E-28	-0.019	0.023	3.99E-01
57	rs11690644	2	102914214	A	0.95	135.12	0.164	0.014	3.12E-31	0.034	0.029	2.51E-01
58	rs962993	10	9053132	C	0.75	153.18	0.117	0.009	3.49E-35	-0.042	0.020	3.57E-02

|

Table S4. Description of Mendelian randomisation methods.

MR method	Description	Assumptions of pleiotropy	Reference
**Inverse Variance Weight - Fixed Effects**	Calculates the ratio between the change of the outcome per unit of exposure to the change in the exposure per unit of change in the instrumental variable (Wald ratio)	IVW-FE assumes no presence of pleiotropy (vulnerable to presence of pleiotropy)	Burgess S, Small DS, Thompson SG. A review of instrumental variable estimators for Mendelian randomization. Stat Methods Med Res. 2017 Oct;26(5):2333-2355. doi: 10.1177/0962280215597579. Epub 2015 Aug 17. PMID: 26282889; PMCID: PMC5642006.
**Inverse Variance Weight - Random Effects**	Calculates the ratio between the change of the outcome per unit of exposure to the change in the exposure per unit of change in the instrumental variable (Wald ratio).<br>Assumes that the pleiotropy is balanced between the genetic instruments (Random Effects) and that the effect is independent of the magnitude of the effect of each genetic instrument.	IVW-RE is robust to pleiotropy if two conditions are met: i) Pleiotropy is balanced (i.e., the mean is zero) and ii) INSIDE assumption (i.e. association between genetic instrument and outcome is not correlated to the path from the genetic instrument to the outcome)	Jack Bowden and others, Improving the accuracy of two-sample summary-data Mendelian randomization: moving beyond the NOME assumption, *International Journal of Epidemiology*, Volume 48, Issue 3, June 2019, Pages 728–742, https://doi.org/10.1093/ije/dyy258
**Weighted Mode-based estimation**	This method assumes that the most frequent causal effect is consistent with the true causal effect.	Even if the instruments outside this effect are not valid, the causal effect is not invalid	Hartwig FP, Davey Smith G, Bowden J. Robust inference in summary data Mendelian randomization via the zero modal pleiotropy assumption. International Journal of Epidemiology 2017;46:1985-1998.
**Weighted median analysis**	A Mendelian Randomization method that obtains an estimator equal to the median of the distribution of all the instruments.	The weighted median estimator will be resistant to pleiotropy if there is at least 50% of the weight of the distribution that is provided by valid instruments	Bowden, J., Davey Smith, G., Haycock, P.C. and Burgess, S. (2016), Consistent Estimation in Mendelian Randomization with Some Invalid Instruments Using a Weighted Median Estimator. Genet. Epidemiol., 40: 304-314. https://doi.org/10.1002/gepi.21965
**MR-Egger regression + SIMEX method**	MR method suitable for situations where not all the genetic instruments satisfy the instrumental variable assumptions	Provides a consistent estimate and detects the presence of directional pleiotropy	Bowden J, Del Greco M F, Minelli C, Davey Smith G, Sheehan NA, Thompson JR. Assessing the suitability of summary data for two-sample Mendelian randomization analyses using MR-Egger regression: the role of the I2 statistic. Int J Epidemiol. 2016;45(6):1961-1974. https://doi:10.1093/ije/dyw220
**Pleiotropy correction**			
**MR-PRESSO**	Evaluates horizontal pleiotropy in a set of multiple genetic instruments (SNPs) within a genome-wide summary association statistics dataset. It detects horizontal pleiotropy with a global test, then corrects for horizontal pleiotropy by removing pleiotropic SNPs and testing whether the results are distorted by the removal or inclusion of the pleiotropic SNPs		https://github.com/rondolab/MR-PRESSO

|

Table S5. Regression estimates for the association between asthma and incident coronary heart disease (CHD)

	Univariable regression estimates			Multivariable, not adjusted by GP-consultation variable			Multivariable, adjusted by GP- consultation variable		
	**Unadjusted HR**	**p-value**	**95% CI**	**Adjusted HR**	**p-value**	**95% CI**	**Adjusted HR**	**p-value**	**95% CI**
**Asthma**	1.14	<0.001	1.09-1.19	1.06	0.033	1.01-1.13	0.99	0.758	0.94-1.05
**OCS category**									
None		*Reference*			*Reference*			*Reference*	
1 OCS	1.45	<0.001	1.27-1.66	1.31	<0.001	1.14-1.52	1.28	<0.001	1.10-1.48
> 1 OCS	1.48	<0.001	1.29-1.71	1.35	<0.001	1.15-1.57	1.26	0.004	1.08-1.48
**Socioeconomic Status (IMD)**									
1 (Least deprived)		*Reference*			*Reference*			*Reference*	
2	1.13	0.030	1.01-1.27	1.08	0.177	0.97-1.21	1.08	0.182	0.95-1.20
3	1.28	<0.001	1.14-1.44	1.16	0.014	1.03-1.31	1.15	0.019	1.02-1.30
4	1.58	<0.001	1.39-1.79	1.41	<0.001	1.24-1.60	1.40	<0.001	1.23-1.59
5 (Most deprived)	1.88	<0.001	1.64.2.15	1.61	<0.001	1.39-1.85	1.57	<0.001	1.37-1.81
**Body Mass Index**									
Normal		*Reference*			*Reference*			*Reference*	
Underweight	1.50	0.002	1.16-1.95	1.37	0.024	1.04-1.79	1.37	0.022	1.05-1.79
Overweight	1.07	0.154	0.98-1.17	1.09	0.088	0.99-1.19	1.08	0.105	0.98-1.19
Obese	1.30	<0.001	1.19-1.44	1.31	<0.001	1.19-1.45	1.28	<0.001	1.16-1.42
**Atopy**									
Yes	1.00	0.918	0.93-1.09	0.98	0.613	0.89-1.07	0.96	0.330	0.88-1.04
**Smoking status**									
Non-smoker (reference)		*Reference*			*Reference*			*Reference*	
Ex-smoker	1.35	<0.001	1.24-1.47	1.31	<0.001	1.19-1.42	1.29	<0.001	1.19-1.41
Smoker	2.20	<0.001	2.00-2.42	2.14	<0.001	1.95-2.36	2.13	<0.001	1.93-2.34
**GP consultation**									
1st quartile		*Reference*						*Reference*	
2nd quartile	1.23	<0.001	1.12-1.35				1.22	<0.001	1.11-1.34
3rd quartile	1.40	<0.001	1.27-1.54				1.33	<0.001	1.20-1.48
4th quartile	1.85	<0.001	1.67-2.05				1.70	<0.001	1.52-1.89

BMI= body mass index, IMD = index of multiple deprivation, GP = general practitioner, HR = hazard ratio. Each model is adjusted for all shown variables in that model.

|

Table S6. Multivariable regression estimates for model 2 including asthma severity

	Univariable regression estimates			Multivariable, not adjusted by GP-consultation variable			Multivariable, adjusted by GP-consultation variable		
	**Unadjusted HR**	**p-value**	**95% CI**	**Adjusted HR**	**p-value**	**95% CI**	**Adjusted HR**	**p-value**	**95% CI**
**Therapy**									
No asthma		*Reference*			*Reference*			*Reference*	
No treatment	0.99	0.917	0.89-1.10	0.97	0.605	0.87-1.09	0.92	0.139	0.83-1.03
SABA only	1.15	0.026	1.02-1.29	1.06	0.368	0.93-1.20	0.99	0.899	0.87-1.13
ICS	1.12	0.002	1.04-1.24	1.08	0.061	0.99-1.17	1.00	0.854	0.93-1.09
ICS + add-on	1.27	<0.001	1.17-1.39	1.13	0.019	1.02-1.25	1.01	0.797	0.91-1.13
**OCS**									
None		*Reference*			*Reference*			*Reference*	
1 OCS	1.45	<0.001	1.27-1.66	1.28	<0.001	1.10-1.49	1.26	0.003	1.08-1.46
> 1 OCS	1.48	<0.001	1.29-1.71	1.29	0.002	1.10-1.52	1.24	0.011	1.05-1.46
**Socioeconomic Status (IMD)**									
1 (Least deprived)		*Reference*						*Reference*	
2	1.13	0.030	1.01-1.27	1.08	0.175	0.97-1.21	1.08	0.180	0.96-1.21
3	1.28	<0.001	1.14-1.44	1.16	0.014	1.03-1.31	1.15	0.018	1.03-1.31
4	1.58	<0.001	1.39-1.79	1.41	<0.001	1.24-1.60	1.41	<0.001	1.24-1.59
5 (Most deprived)	1.88	<0.001	1.64.2.15	1.61	<0.001	1.40-1.85	1.58	<0.001	1.37-1.81
**Body Mass Index**									
Normal		*Reference*			*Reference*			*Reference*	
Underweight	1.50	0.002	1.16-1.95	1.36	0.025	1.04-1.79	1.37	0.022	1.05-1.79
Overweight	1.07	0.154	0.98-1.17	1.08	0.091	0.99-1.19	1.08	0.107	0.98-1.19
Obese	1.30	<0.001	1.19-1.44	1.31	<0.001	1.18-1.45	1.29	<0.001	1.16-1.42
**Atopy**	1.00	0.918	0.93-1.09	0.98	0.594	0.89-1.06	0.96	0.322	0.88-1.04
**Smoking status**									
Non-smoker (reference)		*Reference*			*Reference*			*Reference*	
Ex-smoker	1.35	<0.001	1.24-1.47	1.30	<0.001	1.19-1.42	1.29	<0.001	1.18-1.41
Smoker	2.20	<0.001	2.00-2.42	2.14	<0.001	1.94-2.36	2.13	<0.001	1.93-2.34
**GP consultation**									
1st quartile		*Reference*						*Reference*	
2nd quartile	1.23	<0.001	1.12-1.35				1.22	<0.001	1.11-1.34
3rd quartile	1.40	<0.001	1.27-1.54				1.33	<0.001	1.19-1.47
4th quartile	1.85	<0.001	1.67-2.05				1.69	<0.001	1.51-1.89

|

Table S7. The influence of asthma severity on the association between asthma and incident CHD

Asthma severity	Adjusted HR	95% CI	p-value
Asthma + no treatment	0.91	0.81-1.02	0.102
Asthma + SABA only	0.99	0.86-1.14	0.884
Asthma + ICS	1.00	0.91-1.09	0.981
Asthma + ICS + add-on	1.04	0.92-1.18	0.466

Model 2 stratified by asthma severity. SABA=short-acting beta agonist. ICS=inhaled corticosteroid.

|

Table S8. Distribution according to quantiles of GP consultation variable

	GP consultation quartile										
	1		2		3		4		Total		
	N	%	N	%	N	%	N	%	N	%	Mean standardised difference (1st-4th quantile)
**Exposed**											0.83
No	346,642	45.5%	167,228	22.0%	141,266	18.6%	106,319	14.0%	761,455	100.0%	
Yes	161,035	21.1%	146,942	19.3%	202,850	26.6%	250,628	32.9%	761,455	100.0%	
**Age, years, mean (SD)**	38.3	16.67	37.9	17.11	39.0	17.37	42.6	18.01	39.4	17.34	-0.243
**Sex**											
Male	274,949	41.5%	137,571	20.7%	134,246	20.2%	116,462	17.6%	663,228	100.0%	
Female	232,728	27.1%	176,599	20.5%	209,870	24.4%	240,485	28.0%	859,682	100.0%	
**Index of Multiple Deprivation**											0.18
1 (Least deprived)	112,818	36.0%	67,584	21.6%	71,160	22.7%	61,759	19.7%	313,321	100.0%	
2	105,080	34.6%	65,318	21.5%	69,396	22.8%	64,341	21.2%	304,135	100.0%	
3	97,275	33.3%	60,262	20.7%	66,723	22.9%	67,445	23.1%	291,705	100.0%	
4	99,040	31.4%	64,437	20.4%	72,489	23.0%	79,644	25.2%	315,610	100.0%	
5 (most deprived)	92,906	31.3%	56,271	19.0%	64,061	21.6%	83,464	28.1%	296,702	100.0%	
**BMI**											0.22
Normal	139,111	30.3%	102,037	22.3%	113,253	24.7%	104,010	22.7%	458,411	100.0%	
Underweight	15,134	31.1%	10,669	21.9%	11,229	23.1%	11,640	23.9%	48,672	100.0%	
Overweight	147,781	30.1%	101,804	20.8%	117,653	24.0%	123,222	25.1%	490,460	100.0%	
Obese	63,759	24.9%	46,792	18.3%	60,142	23.5%	85,304	33.3%	255,997	100.0%	
**Smoking status**											0.21
Never	185,738	33.3%	125,458	22.5%	130,777	23.4%	116,513	20.9%	558,486	100.0%	
Ex-smoker	128,873	27.9%	92,116	19.9%	112,041	24.2%	129,571	28.0%	462,601	100.0%	
Smoker	121,828	30.3%	80,839	20.1%	92,794	23.1%	107,079	26.6%	402,540	100.0%	
**Atopy**											0.37
Yes	113,045	24.5%	91,142	19.7%	118,416	25.6%	139,231	30.1%	461,834	100.0%	
**Diabetes**											0.37
Yes	7,526	11.4%	7,962	12.1%	14,501	22.0%	35,965	54.5%	65,954	100.0%	
**Hypertension**											0.32
Yes	28,427	20.0%	24,222	17.0%	34,439	24.2%	55,092	38.7%	142,180	100.0%	
**OCS**											0.43
None	494,360	34.9%	299,128	21.1%	318,189	22.4%	306,162	21.6%	1,417,839	100.0%	
1 OCS	9,154	14.0%	10,405	15.9%	17,392	26.5%	28,664	43.7%	65,615	100.0%	
>1 OCS	4,163	10.6%	4,637	11.8%	8,535	21.6%	22,121	56.1%	39,456	100.0%	
**Therapy**											0.90
No asthma	346,642	45.5%	167,228	22.0%	141,266	18.6%	106,319	14.0%	761,455	100.0%	
No treatment	64,977	27.7%	51,282	21.9%	60,007	25.6%	58,139	24.8%	234,405	100.0%	
SABA only	28,961	21.0%	27,904	20.3%	38,153	27.7%	42,768	31.0%	137,786	100.0%	
ICS	48,027	20.1%	46,018	19.3%	65,725	27.6%	78,693	33.0%	238,463	100.0%	
ICS + add-on	19,070	12.6%	21,738	14.4%	38,965	25.8%	71,028	47.1%	150,801	100.0%	

BMI = body mass index, IMD = index of multiple deprivation (socioeconomic status)

Table S9. Interaction analyses in the observational analysis

Interaction term	Model*	p-value
Smoking (never or ever)	1	0.372
	2	0.191
Sex (females or males)	1	**<0.001**
	2	**<0.001**
Eosinophils (normal <0.3cells/μL or high ≥0.3cells/μL)	1	0.611
	2	0.753
GP consultations (top quartile: yes/no)	1	0.921
	2	0.742

|

Table S9. Sensitivity analysis: association between asthma and coronary heart disease using one-sample MR (UKB only)

Method	Causal effect estimates			Heterogeneity evaluation
	OR	95% CI	p-value	I^2 % (95%CI); P-het Q test
**IVW-FE**	1.012	0.971- 1.055	0.569	46 (28-60); 4.3x10-5
**IVW-FE***	0.995	0.955- 1.037	0.819	20 (0-42); 0.0929
**IVW-RE**	1.012	0.956- 1.071	0.676	
**IVW-RE***	0.995	0.949- 1.043	0.837	
**MR-Egger**	1.033	0.886-1.204	0.681	I^2gx=0.92
**MR-Egger***	1.032	0.910-1.171	0.620	I^2gx=0.93
**WMM**	1.005	0.944-1.070	0.886	
**WMM***	1.002	0.941-1.067	0.942	
**WBM**	0.992	0.921-1.069	0.843	
**WBM***	0.993	0.907-1.087	0.886	

* Excluding 1 pleiotropic instrument (rs112502960)

|

Table S10. MR analyses of the association between asthma and coronary heart disease, stratified by sex, in UKB

Method	Causal effect estimates			Heterogeneity evaluation
	OR	95% CI	p-value	I^2 % (95%CI); P-het Q test
IVW-FE: (Female)	1.023	0.971- 1.055	0.569	20 (0-43); 0.100
IVW-FE: (Male)	1.004	0.958- 1.052	0.877	39 (15-57); 2.5x10-3
IVW-RE (Female)	1.023	0.933- 1. 122	0.633	
IVW-RE (Male)	1.004	0.947- 1.065	0.904	
MR-Egger (Female)	0.979	0.732-1.309	0.886	I^2gx=0.83
MR-Egger (Male)	1.100	0.934-1.294	0.258	I^2gx=0.88
WMM (Female)	1.025	0.901-1.667	0.701	
WMM (Male)	1.014	0.945-1.088	0.689	
WBM (Female)	1.121	0.853-1.472	0.412	
WBM (Male)	1.010	0.916-1.114	0.843	

Figure S1. Flow diagram of the study population

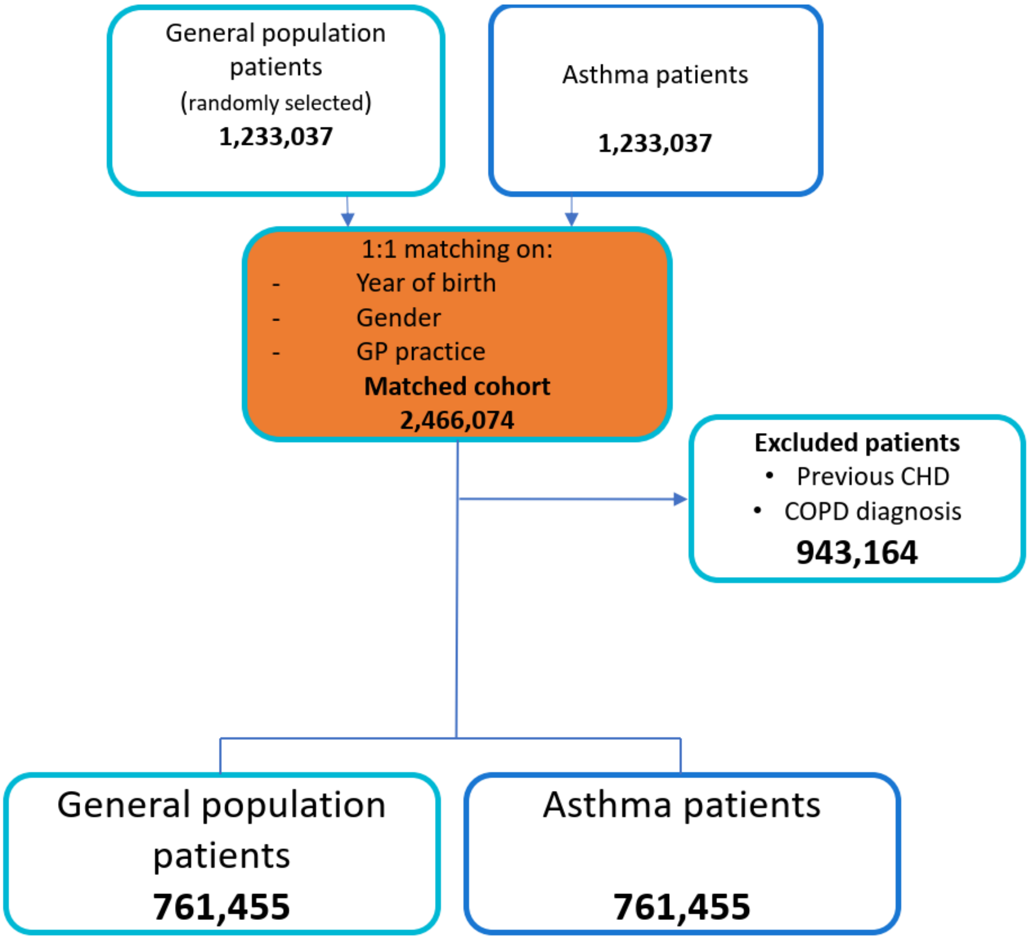

|

Figure S2. Directed acyclic graphs displaying the causal paths between asthma and incident coronary heart disease

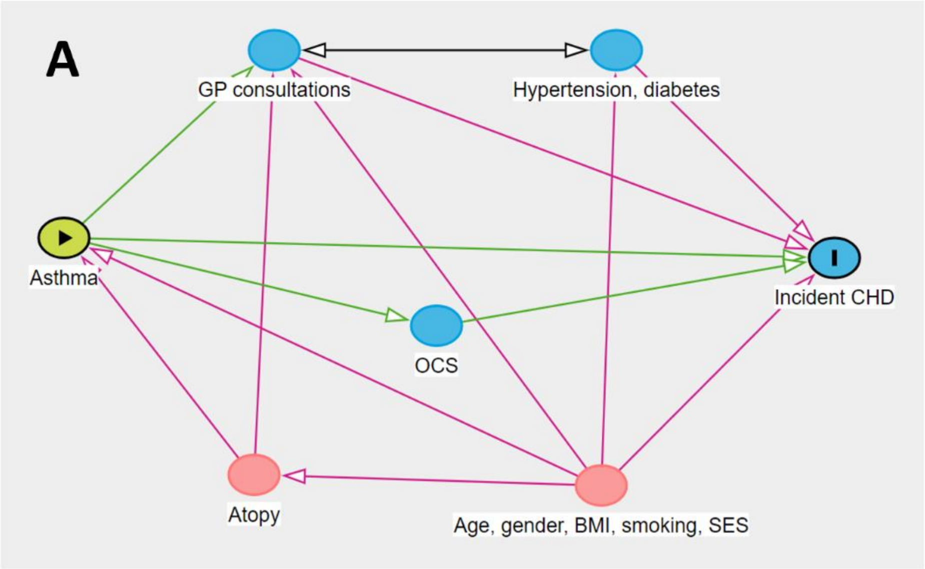

Figure A. Directed acyclic graph (DAG) displaying the path between asthma and incident CHD and potential confounders. GP consultations are considered most likely to be confounding the association. Minimal sufficient adjustment includes all nodes except hypertension/diabetes.

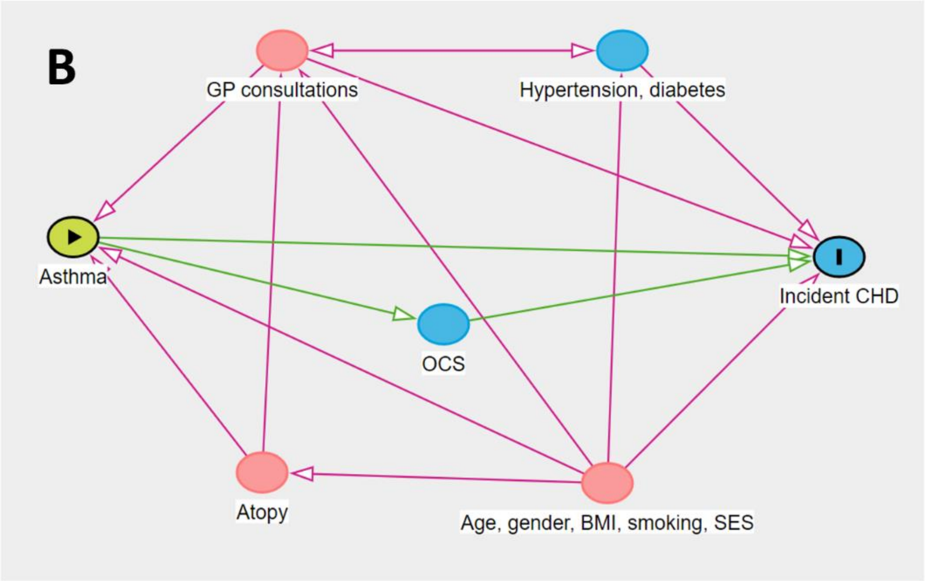

Figure B. DAG displaying the path between asthma and incident CHD if considering GP consultations to be a mediator in the path. As we are only interested in the direct effect we still wish to adjust for the variable even if considered a mediator, not confounder.

CHD = coronary heart disease. SES = socioeconomic status. BMI = body mass index. OCS = oral corticosteroids. DAGs were generated online (http://www.DAGitty.net).

Figure S3. Schematic overview of Mendelian randomisation

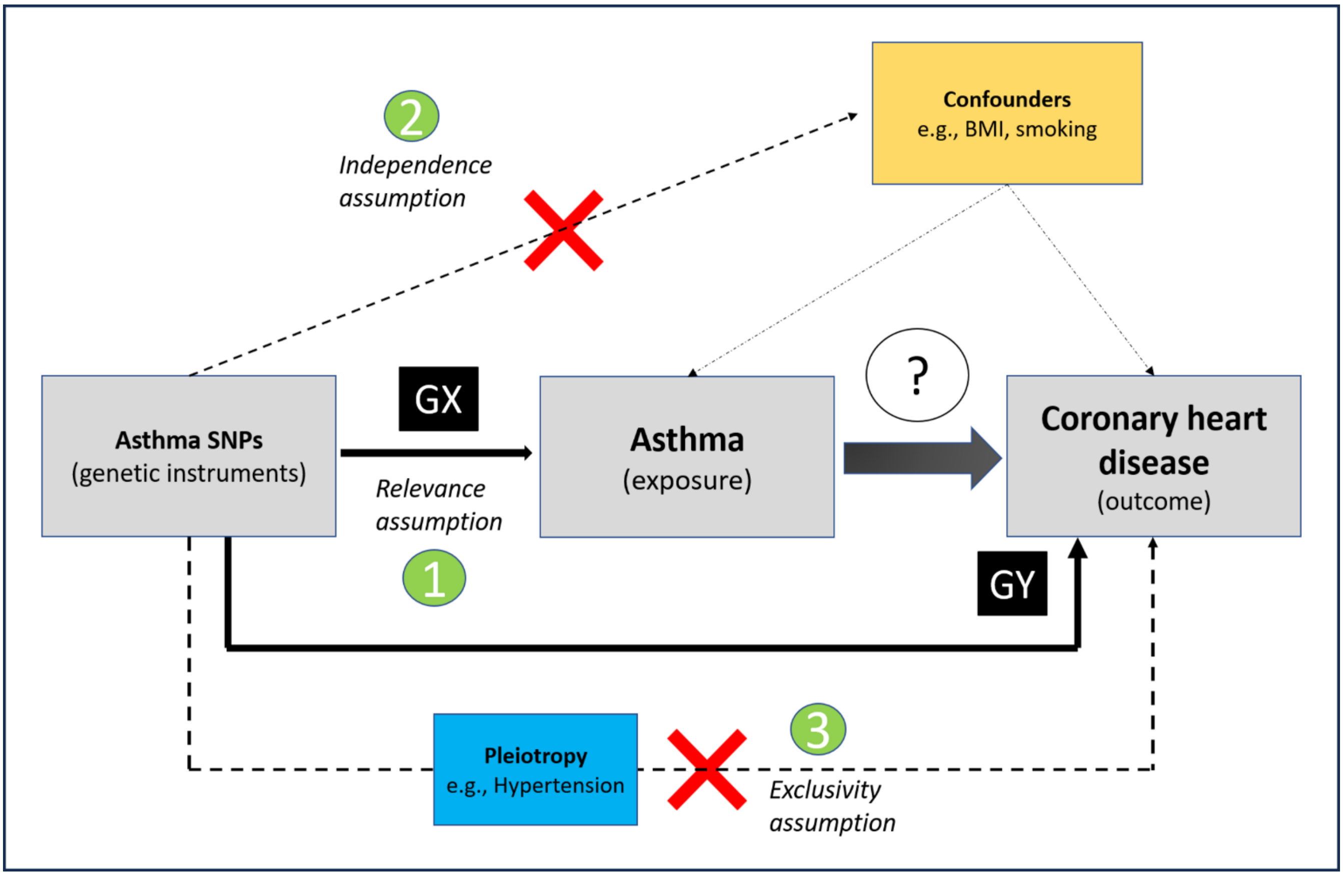

For each single nucleotid polymorphism (SNP), the MR effect estimate is calculated using the GX (association of the SNP with asthma) and GY (association of the SNP with coronary heart disease). Assumptions of MR: 1) SNPs are associated with asthma (exposure) 2) the association between the SNP and the outcome is not confounded. 3) The association between the SNP and outcome is not explained by alternative pathways other than the exposure (pleiotropy).

|

Figure S4. Risk of CHD events in asthma and in the general population

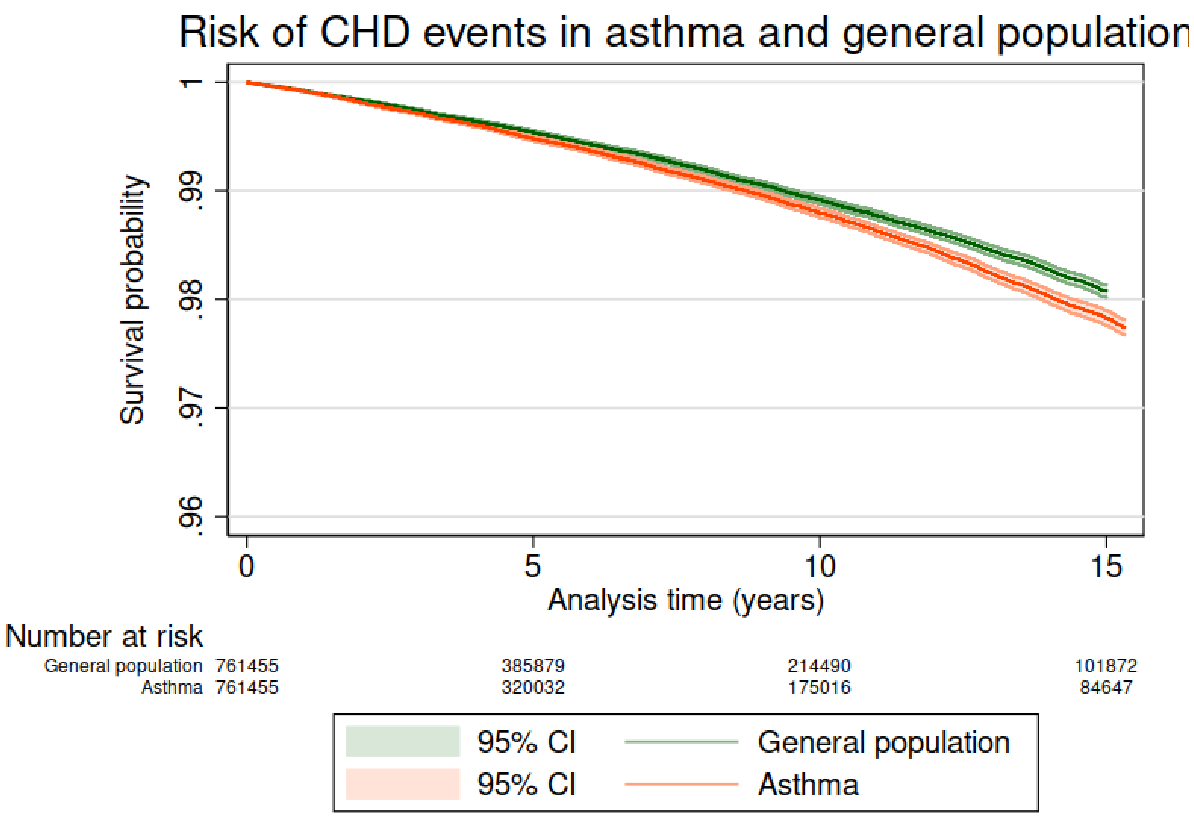

|

Figure S5. Association between asthma and minor ailments

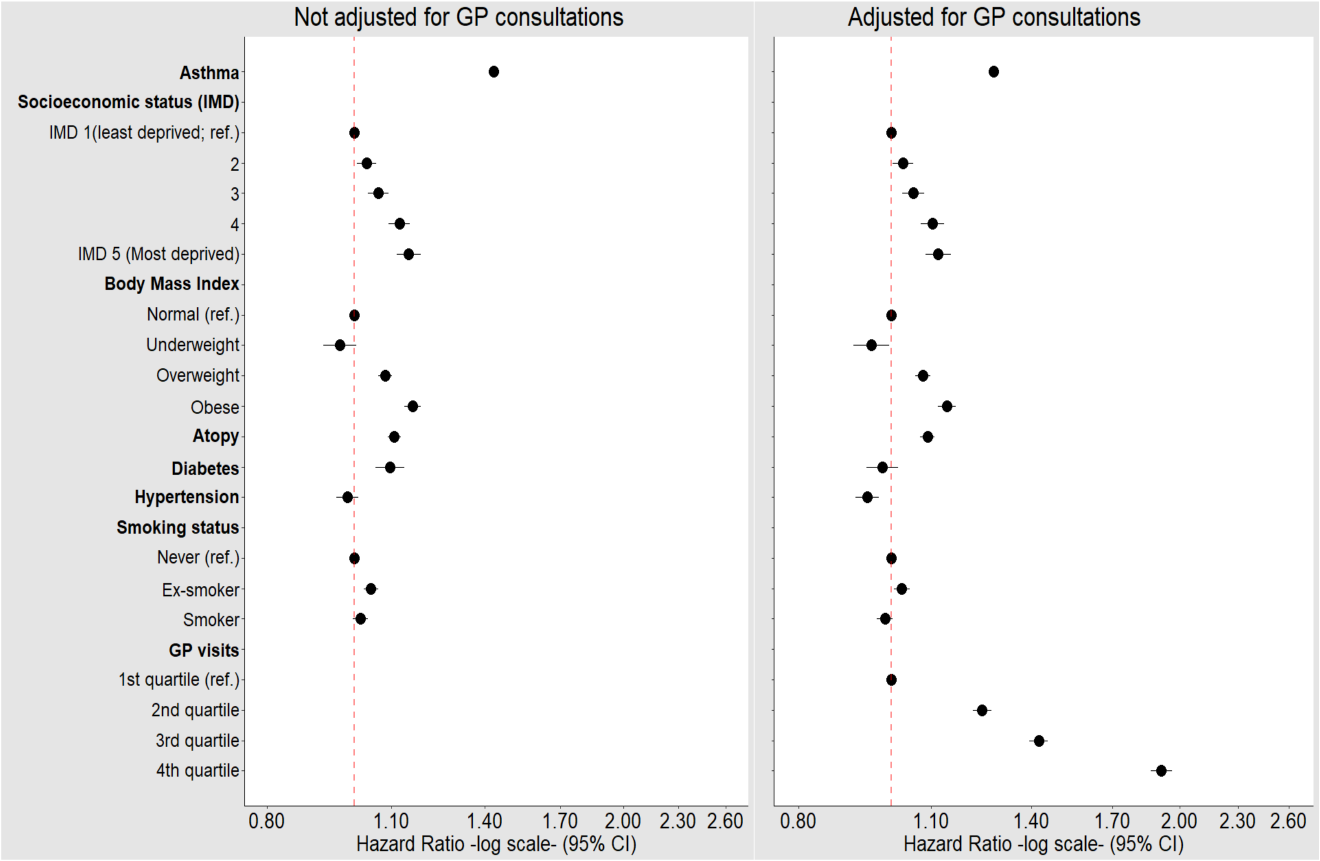

|

Figure S6. Association between asthma and all-cause mortality

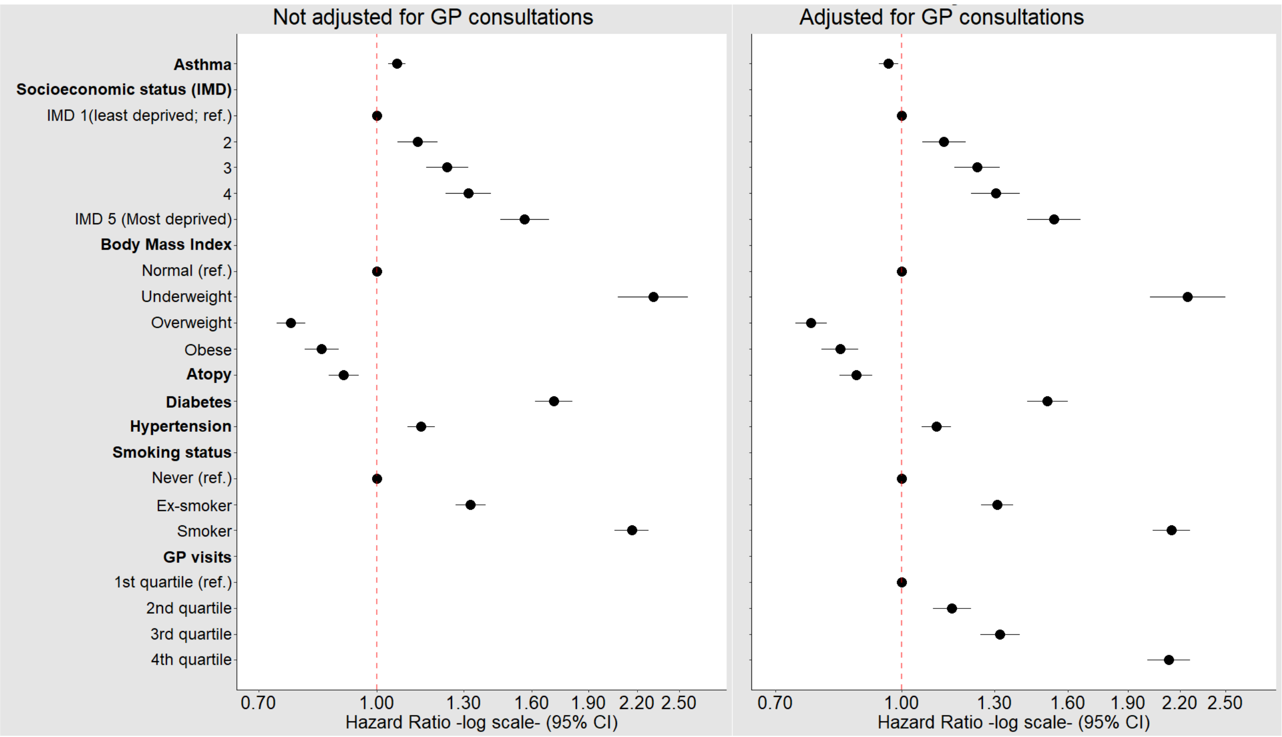

|

Figure S7. Association between asthma and composite CHD outcome, stratified by sex

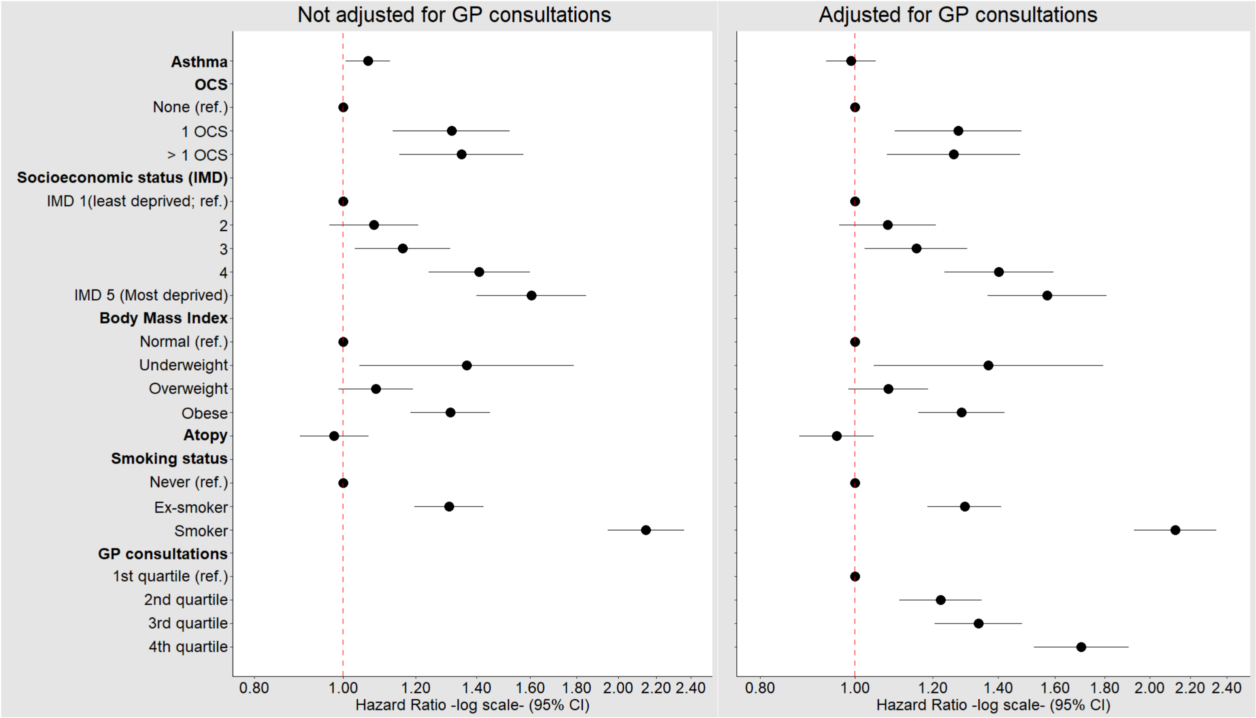

|

Figure S8. Individual variant contributions to Cochran's Q heterogeneity statistic with the 5th (dotted line) and Bonferroni corrected (0.05/62th) percentiles (dashed line) of a chi-squared with 1 degree of freedom.

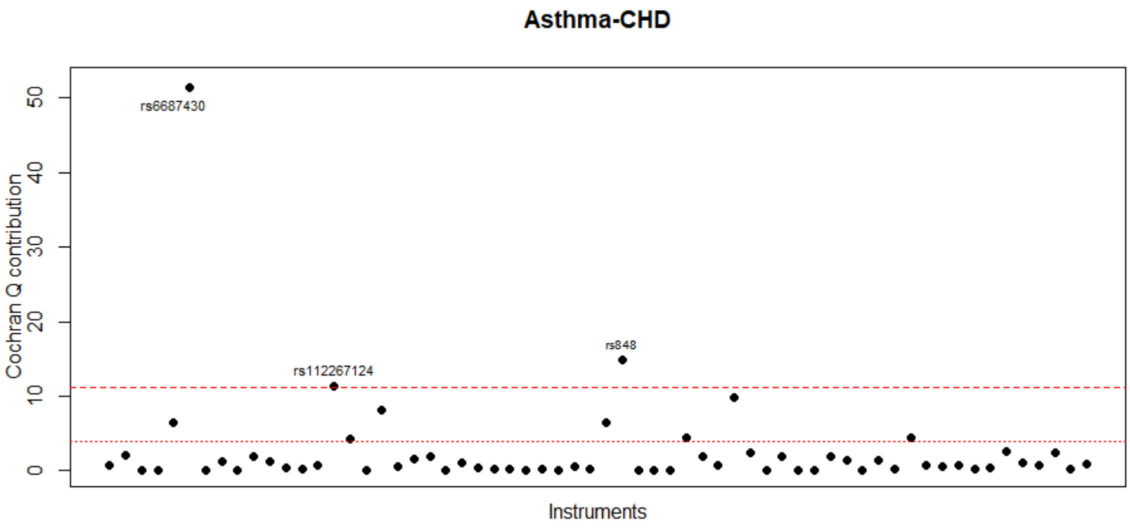

Supplement: Supplementary file 1 [file ERJ-01788-2023.SUPPLEMENT.pdf]
