# Supplementary material for: Asthma and incident coronary heart disease: an observational and Mendelian randomisation study
Source: Eur Respir J. 2023 Nov 30;62(5):2301788. doi: 10.1183/13993003.01788-2023 (PMC10695770; doi:10.1183/13993003.01788-2023)
Supplement: Supplementary file 2 [file ERJ-01788-2023.Shareable.pdf]

SHAREABLE PDF

Asthma and incident coronary heart disease: an observational and Mendelian randomisation study

Carlos A. Valencia-Hernández [1], Fabiola Del Greco M [2], Varun Sundaram [3], Laura Portas [1,4], Cosetta Minelli [1] and Chloe I. Bloom [1]

[1]National Heart and Lung Institute, Imperial College London, London, UK. [2]Institute for Biomedicine, Eurac Research, Bolzano, Italy. [3]Louis Stokes Cleveland Medical Center, Case Western Reserve University, Cleveland, OH, USA. [4]Oxford Big Data Institute, University of Oxford, Oxford, UK.

Corresponding author: Chloe I. Bloom (chloe.bloom06@imperial.ac.uk)

Check for updates

Shareable abstract (@ERSpublications)
Our study using a triangulation approach, real-world data and genetic epidemiology found no association between asthma and coronary heart disease, in contrast to earlier studies https://bit.ly/3FuZHaP

Cite this article as: Valencia-Hernández CA, Del Greco M F, Sundaram V, *et al*. Asthma and incident coronary heart disease: an observational and Mendelian randomisation study. *Eur Respir J* 2023; 62: 2301788 [DOI: 10.1183/13993003.01788-2023].

This extracted version can be shared freely online.

Copyright ©The authors 2023.

This version is distributed under the terms of the Creative Commons Attribution Licence 4.0.

This article has an editorial commentary: https://doi.org/10.1183/13993003.02009-2023

Received: 1 Sept 2023
Accepted: 23 Oct 2023

Abstract

Background Observational studies suggest asthma is a risk factor for coronary heart disease (CHD) and sex modifies the risk, but they may suffer from methodological limitations. To overcome these, we applied a "triangulation approach", where different methodologies, with different potential biases, were leveraged to enhance confidence in findings.

Methods First, we conducted an observational study using UK medical records to match asthma patients 1:1, by age, sex and general practitioner (GP) practice, to the general population. We measured the association between asthma and incident CHD (myocardial infarction: hospitalisation/death) by applying minimal sufficient adjustment: model 1, smoking, body mass index, oral corticosteroids, atopy and deprivation; model 2, additionally adjusting for healthcare behaviour (GP consultation frequency). Second, we conducted a Mendelian randomisation (MR) study using data from the UK Biobank, Trans-National Asthma Genetic Consortium (TAGC) and Coronary Artery Disease Genome-wide Replication and Meta-analysis consortium (CARDIoGRAM). Using 64 asthma single nucleotide polymorphisms, the effect of asthma on CHD was estimated with inverse variance-weighted meta-analysis and methods that adjust for pleiotropy.

Results In our observational study (n=1 522 910), we found asthma was associated with 6% increased risk of CHD (model 1: HR 1.06, 95% CI 1.01–1.13); after accounting for healthcare behaviour, we found no association (model 2: HR 0.99, 95% CI 0.94–1.05). Asthma severity did not modify the association, but sex did (females: HR 1.11, 95% CI 1.01–1.21; males: HR 0.91, 95% CI 0.84–0.98). Our MR study (n=589 875) found no association between asthma and CHD (OR 1.01, 95% CI 0.98–1.04) and no modification by sex.

Conclusions Our findings suggest that asthma is not a risk factor for CHD. Previous studies may have suffered from detection bias or residual confounding.
